# Effect of preservation on fish morphology over time: Implications for morphological studies

**DOI:** 10.1371/journal.pone.0213915

**Published:** 2019-03-21

**Authors:** V. Alex Sotola, Cody A. Craig, Peter J. Pfaff, Jeremy D. Maikoetter, Noland H. Martin, Timothy H. Bonner

**Affiliations:** Department of Biology/Aquatic Station, Texas State University, San Marcos, Texas, United States of America; DePaul University, UNITED STATES

## Abstract

It has long been recognized that the process of preserving biological specimens results in alterations of body shape, though detailed studies examining the degree to which morphological changes occur throughout the preservation process are lacking. We utilize geometric morphometric analyses, an increasingly common tool for examining shape variation in a wide variety of biological disciplines, to examine the effects of formalin and ethanol preservation on the body shape of 10 freshwater fish species over time: from fresh specimens to eight weeks after preservation. We found significant changes in body shape among fresh and formalin fixed specimens. Furthermore, changes in body shape continue to occur after subsequent ethanol preservation. Two fish species collected at multiple localities show significant morphological differences for a limited number of morphometric characters. However, the significance, or lack thereof, often changed inconsistently from one stage of preservation to another. We conclude that morphometric analyses would ideally be performed on fresh specimens. However, recognizing that this is not always feasible, it is important to be aware of the morphometric changes that can occur during preservation.

## Introduction

Alterations in body shape due to preservation (i.e., most commonly for fish specimens, fixation in 10% formalin then transferred to 70% ethanol for long-term storage) are manifested in a variety of ways [1–2]. Previous studies have found that standard body lengths are shorter after formalin fixation and ethanol preservation [3–7], although fish specimens treated in a 10% formalin solution tend to increase in weight [3,6]. More recently, geometric morphometric analyses have been applied as a tool to measure body shape related to a number of biological questions including speciation, species identification, fish-habitat association, and hybridization [8–11]. Geometric morphometric analyses use landmarks, rather than linear measurements, which are homologous and easily distinguishable points on individuals of the same species, to extract shape information. Such analyses are more effective in analyzing and interpreting body shape and form than several linear or meristic measurements [12]. Many studies have performed geometric morphometric analyses on preserved specimens [10,13,14], and inferences arising from such studies have the potential to be problematic, as alterations in body shape are known to occur during preservation [3,15–20].

Despite the wide usage of preserved specimens, the effects of preservation are often not discussed as a limitation or potential source of error in studies that assess body shape (e.g., [13,21–23]). To obtain a more robust and quantitative assessment of how fish body shapes change after preservation, previous studies have used geometric morphometric analyses [18,20,24]. One study assessed changes in morphology after a total of 90 days of preservation [20], whereas another assessed body shape after both freezing at -20°C and being stored in ethanol for 90 days [18]. Both found significant morphological changes of specimens pre and post-preservation, with the largest changes occurring in the eye and body depth and cautioned against using preserved specimens [18,20]. Another study found no significant effects of formalin fixation and isopropanol preservation time on fish body shape, including specimens which were preserved up to 70 years [24]. However, this study did not test preserved specimens relative to fresh, unpreserved specimens (all specimens were preserved).

The results of previous studies [18,20] were valuable in showing that body shape changes with preservation, however such studies only examined morphological changes at a single time after preservation and not changes in morphology incrementally throughout the preservation process. Additionally, such studies have only assessed changes in morphology due to preservation in a small number of species (i.e. 1–4). Thus, information is lacking in how preservation could affect species across several taxonomic levels (i.e. species, genera, families). Assessing changes in body shape incrementally could provide valuable information if live or recently-deceased organisms are not available for morphometric analysis. If significant morphological change occurs incrementally throughout preservation, it would be advantageous to know the timing and degree to which such changes occur such that morphometric measurements are taken at similar time-periods during preservation.

The purpose of the current study was to document the degree to which changes in body shape due to preservation occur over time in disparately related fish species, and to determine whether morphometric analyses performed at various stages of preservation time might influence any inferences drawn from such analyses. To accomplish this, we used geometric morphometric analyses to address two objectives. Our first objective was to assess changes in body shape among and between 10 species of fishes representing three different families and six genera throughout the preservation process; preserved specimens were examined in two-week increments over eight weeks. Our second objective was to quantify morphological changes in two fish species from different collection locales over an eight-week preservation process.

## Methods

All samples were collected under a Texas Parks and Wildlife Scientific Permit (SPR-0601-159), and study protocols and methods were approved by Texas State University Institutional Animal Care and Committee (1207-0109-01). A total of 10 fish species from disparately-related taxonomic groups were sampled for geometric morphometric assessment: *Cyprinella lutrensis*, *C*. *venusta*, *Macrhybopsis hyostoma*, *M*. *marconis*, *Notropis chalybaeus*, *N*. *amabilis*, *Gambusia geiseri*, *Etheostoma spectabile*, http://txstate.fishesoftexas.org/etheostoma%20fonticola.htm*Percina apristis*, and *P*. *carbonaria*. Sample sizes ranged from six to 74 individuals per species (Table 1). Individuals were captured via seining in several rivers throughout central Texas. Following standard procedures, fishes were euthanized with Tricaine Methanesulfonate (Western Chemical, Inc.). Within one hour of capture and immediately following euthanization, digital photographs were taken with a Nikon D40 digital camera with an 18–55 mm lens mounted on a stand for photograph consistency. Each specimen was pinned into place and photographed on the left-hand side along with a standard metric ruler to provide scale. After the picture was taken, individual specimens were stored upright in individually marked vials and preserved using a 10% formalin solution. Fishes were stored in formalin for two weeks, at which point another picture was taken of each fish, again on the left-hand side along with a metric ruler, and then individuals were transferred to a 70% ethanol solution [25]. Using the same techniques, pictures of each individual were again taken every two weeks, up to eight weeks for a total of five time periods. Time periods will be referred to as field (unpreserved fish), two weeks (2W), four weeks (4W), six weeks (6W), and eight weeks (8W). All photographs were taken by the same two individuals for consistency.

**Table 1:** Species assessed in study, with family and sample sizes (N).

Species	Family	N
*Cyprinella lutrensis*	Cyprinidae	37
*Cyprinella venusta*	Cyprinidae	74
*Macrhybopsis hyostoma*	Cyprinidae	15
*Macrhybopsis marconis*	Cyprinidae	40
*Notropis chalybaeus*	Cyprinidae	6
*Notropis amabilis*	Cyprinidae	14
*Gambusia geiseri*	Poeciliidae	21
*Etheostoma spectabile*	Percidae	18
*Percina apristis*	Percidae	12
*Percina carbonaria*	Percidae	6

Species used to analyze morphological changes over time post-preservation, their respective taxonomic families and sample sizes.

**Fig 1:**
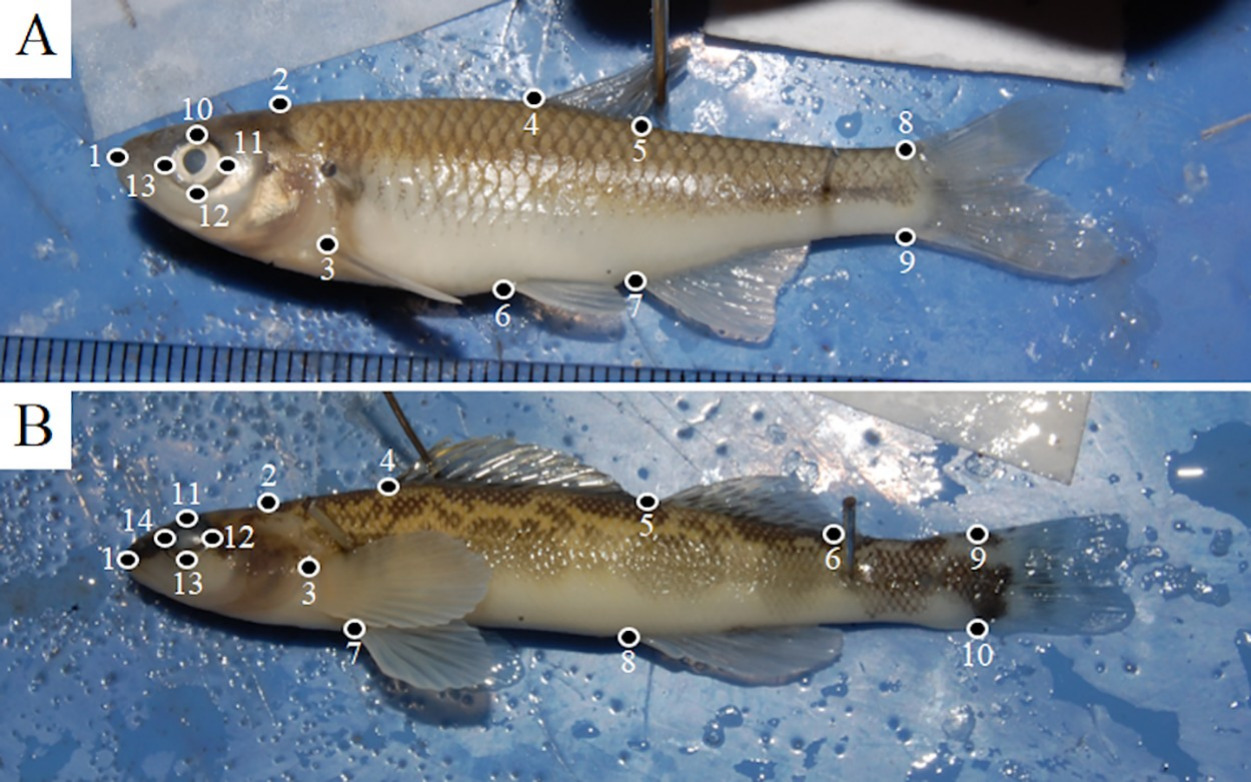
Landmark locations on specimens. Pictures of landmark locations for all non-darters (cyprinids and poeciliids; A) and darters (percids; B). Cyprinids and poeciliids had 13 total landmarks, with percids having 14 total landmarks. Landmarks were placed as follows on cyprinids and poeciliids: (1) snout origin, (2) origin of nape, (3) pectoral fin origin, (4) dorsal fin origin, (5) dorsal fin termination, (6) pelvic fin origin, (7) anal fin origin, (8) upper caudal fin origin, (9) lower caudal fin origin, and (10–13) eye. Landmarks were placed as follows on percids: (1) snout origin, (2) origin of nape, (3) pectoral fin origin, (4) first dorsal fin origin, (5) second dorsal fin origin, (6) second dorsal fin termination, (7) pelvic fin origin, (8) anal fin origin, (9) upper caudal fin origin, (10) lower caudal fin origin, and (11–14) eye.

Thin-plate spline (TPS) files of the images were created with tpsUtil (version 1.70; [26]). Digital landmarks were placed based off the truss system of landmarks [27,28] using tpsDIG (version 2.26; Fig 1; [29]). Landmarks were placed by three individuals, who were trained together to ensure consistency when placing all landmarks. The same researcher placed landmarks on all specimens of one species so that the landmarks of all individuals within a species were consistent across the respective time periods. In all, 13 landmarks were placed on species with one dorsal fin (Cyprinidae and Poeciliidae; Fig 1A), and 14 landmarks were placed on species with two dorsal fins (Percidae; Fig 1B). TPS files containing landmark coordinates were then imported into R [30] for analysis using the package *geomorph* [31].

A generalized Procrustes analysis (GPA) was performed first for each species separately to compute Procrustes coordinates for analysis, and subsequently checked for outliers [32,33]. Landmarks on outlier individuals were re-examined to ensure landmarks were correctly placed, and those individuals which remained outliers were removed from subsequent analyses. Size-dependent variation (allometry) was tested for (using the *procD*.*allometry* function; 10,000 iterations) and removed from the data by obtaining size-adjusted residuals [34]. The size-adjusted residuals were obtained by regressing the Procrustes coordinates on centroid size and obtaining the residuals from the model, which were subsequently used in principal components analysis. Prior to running the final analyses, we compared morphological data where size-dependent variation was removed and where it was not, and no biologically interpretable differences between the two datasets were found. Thus, all subsequent analyses were performed on the dataset where size-dependent variation was removed. The first two principal component axes were plotted to show variation in the data within and between groups (time periods). To test for differences in morphology between time periods, we used the function “repeated_measures_test” from the package *GeometricMorphometricsMix* for each species [35,36,37]. All PC axis coordinates, for species *C*. *venusta*, *C*. *lutrensis*, and *M*. *marconis*, or at least the number of axes which avoided the issue of a singular matrix, for the remaining species, were used in this analysis. With this test we tested for differences between field and 2W, field and 8W, and 2W and 8W; these were chosen to see if there are significant differences in morphology after formalin fixation, overall preservation changes, and after ethanol preservation, respectively. A classical Bonferroni correction was applied for each species. Shape changes associated with PC axes 1 to 5 were visualized and plotted for each species representing individuals that had the minimum and maximum PC value. Shape change plots (mean shape for each time period) were calculated and constructed from the size-adjusted residuals for each time period using the function *shape*.*predictor* [31]. Each time period was plotted relative to field to visualize changes in morphology for each species; a magnification of 1 was used to visualize shape differences.

Centroid sizes, the measurement of overall body size, were calculated as the square root of the sum-squared distances from landmarks to the centroid [38] and were acquired in R with the *geomorph* package. A repeated measures generalized linear model (GLM), using the function “lme”, was performed in R with the *nlme* package to obtain estimates of centroid size by treatment and determine whether centroid sizes change over time [39,40].

Samples of *C*. *lutrensis* and *C*. *venusta* collected from multiple sampling locations were used to quantify morphological differences among sites and across preservation time periods. Both species were chosen because they had sample sizes greater than five individuals for each location (S1 Table). Separate generalized Procrustes analyses were performed on each time period. Subsequently, Procrustes ANOVAs were performed on each time period with 10,000 iterations, and pairwise comparisons were examined to determine how time periods affect morphology with respect to collection locale; alpha values were adjusted with a classical Bonferroni correction. Average Procrustes distances were calculated between sites for each time period for both species [41], and subsequently the percent change for each time period relative to field between each site comparison was calculated. Percent change was calculated as:

$$(\frac{TPi-TP1}{TP1})*100$$

where *TP1* is field Procrustes distance, and *TPi* is each subsequent time period Procrustes distance. Additionally, a Procrustes analysis (function “protest”) was performed on the first two PC axes to statistically compare (with 1,000 permutations) PCA scores of these axes between the different time periods using the *vegan* package in R [42].

## Results

### Morphological differences across preservation time

**Fig 2:**
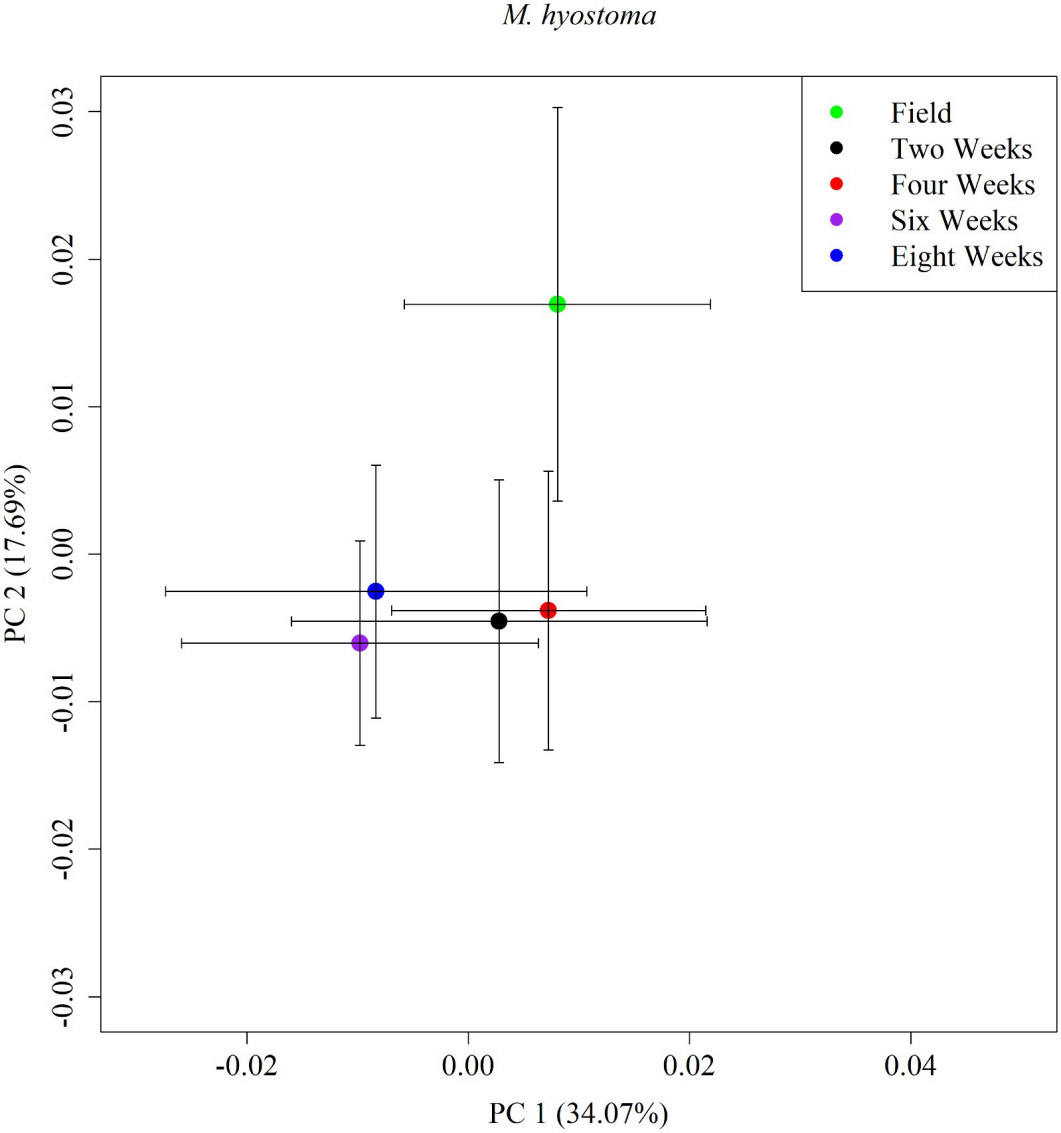
Mean PCA plot of PC axes 1 and 2 for *M*. *hyostoma*. Mean PC plot for PC axes 1 and 2 for *M*. *hyostoma*. Each color represents a different time period during preservation: green is field, black is two weeks, red is four weeks, purple is six weeks, and blue is eight weeks. Error bars is one standard deviation.

The first five PC axes explained between 75 and 87% of the variance among species (Table 2). Seven of 10 species showed a pattern of field body shape separating from all other time periods, such as with *M*. *hyostoma* (Fig 2, S1 Fig).

**Table 2:** Percent variance explained for each of the first five PC axes for each species.

Species	PC1	PC2	PC3	PC4	PC5
*C*. *venusta*	32.81	16.15	12.10	8.44	7.98
*G*. *geiseri*	33.68	16.36	12.65	8.15	7.03
*M*. *hyostoma*	34.07	17.69	11.22	9.32	5.96
*C*. *lutrensis*	26.69	17.24	13.31	11.74	6.39
*M*. *marconis*	38.10	12.70	11.12	8.90	5.44
*N*. *amabilis*	31.10	16.36	13.41	10.66	6.46
*N*. *chalybaeus*	28.98	25.02	16.32	8.51	6.39
*P*. *apristis*	40.99	16.99	12.49	6.70	5.21
*P*. *carbonaria*	33.20	23.49	14.38	10.63	5.19
*E*. *spectabile*	27.19	21.02	10.84	10.19	7.96

Percent variance explained for each of the first five PC axes for each species.

For a majority of the species, curvature of fish (body arching, upward or downward arched dorsal area) was associated with PC1 and the abdominal region (being enlarged or shrunken) was associated with PC1 through PC5. The caudal region (changes in length or width) was associated with PC1 through PC3, head (length or tilt) was associated with PC4 and PC5, and lastly the eye (size) was associated with PC4 (S2 Fig). Repeated measures test found five of 10 species had at least one significant difference (after Bonferroni correction) in morphology between preservation time periods (P < 0.017; Table 3), including three of six cyprinids, the poecilid, and one of three darters. For all but one species, *E*. *spectabile*, all three comparisons were significantly different; for *E*. *spectabile* there was no significant difference between 2W and 8W.

**Table 3:** Repeated measures test output for each species.

Species	Time Period Comparison	Axes	Hotelling’s T^2^	P-value
*C*. *lutrensis*	2W-8W	All	217.615	0.007*
	Field-8W	All	191.564	< 0.001*
	Field-2W	All	319.039	< 0.001*
*C*. *venusta*	2W-8W	All	217.534	< 0.001*
	Field-8W	All	609.523	< 0.001*
	Field-2W	All	154.476	< 0.001*
*M*. *hyostoma*	2W-8W	9	97.533	0.038
	Field-8W	9	53.188	0.135
	Field-2W	9	103.925	0.032
*M*. *marconis*	2W-8W	All	342.784	< 0.001*
	Field-8W	All	368.747	< 0.001*
	Field-2W	All	368.983	< 0.001*
*N*. *amabilis*	2W-8W	9	13.198	0.785
	Field-8W	9	13.189	0.786
	Field-2W	9	35.909	0.332
*G*. *geiseri*	2W-8W	9	112.243	< 0.001*
	Field-8W	9	69.833	0.008*
	Field-2W	9	152.181	< 0.001*
*N*. *chalybaeus*	2W-8W	5	21.184	0.673
	Field-8W	5	4.664	0.932
	Field-2W	5	43.417	0.518
*E*. *spectabile*	2W-8W	9	115.584	0.004*
	Field-8W	9	39.282	0.114
	Field-2W	9	77.035	0.017*
*P*. *apristis*	2W-8W	8	62.815	0.163
	Field-8W	8	26.259	0.462
	Field-2W	8	112.13	0.066
*P*. *carbonaria*	2W-8W	5	7.364	0.875
	Field-8W	5	7.009	0.882
	Field-2W	5	5.434	0.915

Repeated measures test outputs including Hotelling’s T^2^, and p-values for each species testing for differences between time periods (time period comparison) and PC coordinates included for each species (axes). An * indicates a significant difference after Bonferroni correction.

### Centroid size and shape change plots by species

Centroid sizes changed among preservation time periods for all species except *P*. *carbonaria*, indicating body changes over preservation time relative to field specimens (Table 4). Negative slopes among time periods indicated consistent shrinkage between landmarks among cyprinids and poeciliids relative to field specimens (S2 Table). There was some shrinkage, but also enlargement between landmarks for percids relative to field specimens (S3 Table).

**Table 4:** Repeated measures ANOVA output for centroid sizes over time.

Species	num DF	den DF	F-value	P-value
*C*. *lutrensis*	4	144	41.9745	<.0001*
*C*. *venusta*	4	292	19.023	<.0001*
*M*. *hyostoma*	4	56	24.3632	<.0001*
*M*. *marconis*	4	152	29.6581	<.0001*
*N*. *amabilis*	4	52	59.999	<.0001*
*G*. *geiseri*	4	80	66.522	<.0001*
*N*. *chalybaeus*	4	20	32.96	<.0001*
*E*. *spectabile*	4	68	3.4797	0.012*
*P*. *apristis*	4	44	12.6558	<.0001*
*P*. *carbonaria*	4	20	1.084	0.391

Repeated measures ANOVA numerator degrees of freedom (num DF), denominator degrees of freedom (den DF), F-value, and P-values testing significance of GLM models for a change in centroid size for all species throughout the time periods. An * indicates a significant difference.

**Fig 3:**
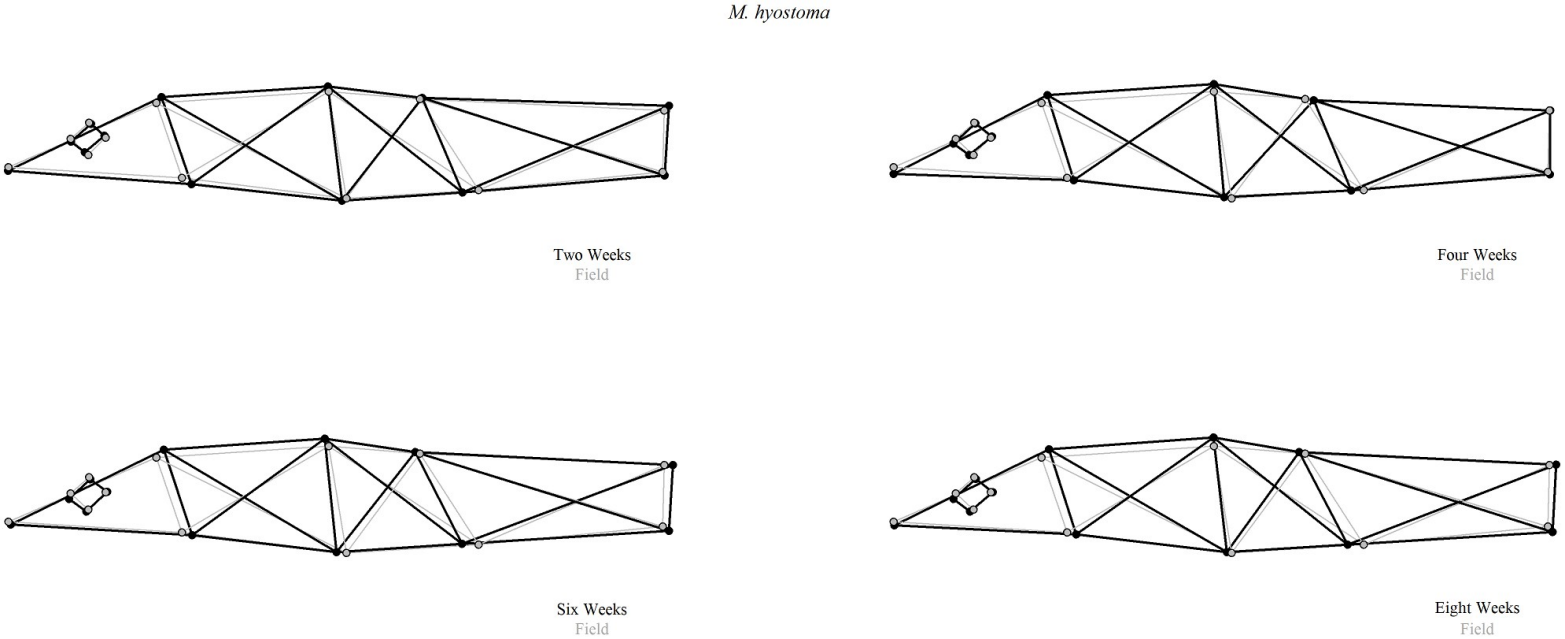
Shape change plot. Example of how landmarks of preserved samples shifted relative to field samples in a shape change plot of *M*. *hyostoma*. Gray points and lines represent the field time period, with black points and lines representing each other time period (2W through 8W).

With specific landmarks in shape change plots, trends in body shape changes were relatively consistent across species. There was some shrinkage but also corresponding enlargement between landmarks. Trends in shape change plots progressing from field to 8W time period were an increase in abdomen girth, decrease in abdomen length, and an initial decrease in caudal peduncle length in 2W followed by an increase through 8W. Smaller changes were observed with expanding of eye size and lengthening of the head region from field to 8W (Fig 3, S3 Fig).

### Morphological changes among sites

Body shapes differed (P <0.05) among collection locales for *C*. *lutrensis* and *C*. *venusta* for all preservation time periods examined (Table 5). For the field time period of *C*. *lutrensis*, morphology differed between five of six pairwise site comparisons. After two weeks in formalin, morphology differed between one of six of the *C*. *lutrensis* pairwise site comparisons. After switching to ethanol storage, morphology differed between three pairwise site comparisons at 4W and 6W, and five pairwise site comparisons at 8W time periods (Table 6).

**Table 5:** Procrustes ANOVA testing differences by site within time periods.

Species	Time	N_DF_	D_DF_	F-value	P-value
*C*. *venusta*	Field	6	67	5.4398	0.001*
	2W	6	67	4.4958	0.001*
	4W	6	67	3.3858	0.001*
	6W	6	67	5.5722	0.001*
	8W	6	67	5.2467	0.001*
*C*. *lutrensis*	Field	3	30	3.492	0.001*
	2W	3	30	0.5595	0.001*
	4W	3	30	3.5533	0.001*
	6W	3	30	4.093	0.001*
	8W	3	30	3.1127	0.001*

Procrustes ANOVA (F-value and P-values) testing for differences between sites for *C*. *lutrensis* and *C*. *venusta* within each preservation time-period. Field represents measurements taken on freshly collected field specimens, followed by preservation of two weeks (2W) through eight weeks (8W). An * indicates a significant difference.

**Table 6:** Pairwise comparisons of Procrustes ANOVA for *C*. *lutrensis*.

Field	Academy	Cuero	Goliad
Cuero	0.265		
Goliad	0.001*	0.001*	
Gonzalez	0.006*	0.004*	0.001*
Two Weeks	Academy	Cuero	Goliad
Cuero	0.013		
Goliad	0.001*	0.023	
Gonzalez	0.016	0.366	0.108
Four Weeks	Academy	Cuero	Goliad
Cuero	0.002*		
Goliad	0.001*	0.073	
Gonzalez	0.001*	0.515	0.059
Six Weeks	Academy	Cuero	Goliad
Cuero	0.001*		
Goliad	0.001*	0.117	
Gonzalez	0.001*	0.632	0.064
Eight Weeks	Academy	Cuero	Goliad
Cuero	0.001*		
Goliad	0.001*	0.001*	
Gonzalez	0.007*	0.501	0.003*

Pairwise Procrustes ANOVA comparison P-values for *C*. *lutrensis* by site within each time period, from field to eight weeks.

*indicates a significant difference after Bonferroni correction.

The highest Procrustes correlations were found between 4W and 8W for *C*. *lutrensis*, and the lowest Procrustes correlations were between field and 2W, 4W, 6W, 8W (Table 7).

**Table 7:** Procrustes correlations of PCA ordination scores for *C*. *lutrensis* (bottom half) and *C*. *venusta* (top half).

	Field	Two	Four	Six	Eight
Field		0.428	0.537	0.506	0.556
Two	0.341		0.886	0.891	0.861
Four	0.439	0.792		0.939	0.922
Six	0.274	0.551	0.791		0.876
Eight	0.442	0.775	0.819	0.646	

Procrustes correlations of PCA plots for *C*. *lutrensis* (bottom half) and *C*. *venusta* (top half). Field represents measurements taken on freshly dead specimens, followed by preservation of two weeks (2W) through eight weeks (8W). All comparisons were significant at the 0.05 level.

For the field time period of *C*. *venusta*, morphology differed between 10 of 21 pairwise site comparisons. After two weeks in formalin, morphology differed between eight pairwise site comparisons. At the 4W preservation period, morphology differed between 10 pairwise site comparisons, nine at 6W, and nine at 8W (Table 8). Lastly, the highest Procrustes correlations were found between 4W and 6W, 8W for *C*. *venusta*, and the lowest Procrustes correlations were between field and 2W, 4W, 6W, 8W (Table 7). There were also changes in Procrustes distances between pairwise site comparisons across time periods (S4 and S5 Tables). For *C*. *lutrensis*, on average, the highest absolute percent change in Procrustes distances occurred after six weeks of preservation relative to field, followed by four weeks, two weeks, then eight weeks of preservation (S6 Table). For *C*. *venusta*, six weeks also had the highest absolute percent change in Procrustes distances relative to field, though two weeks was second highest, followed by eight weeks then four weeks (S7 Table).

**Table 8:** Pairwise comparisons of Procrustes ANOVA for *C*. *venusta*.

Field	Academy	Bandera	Comfort	Driftwood	Easterly	Kempner
Bandera	0.011					
Comfort	0.001*	0.292				
Driftwood	0.001*	0.001*	0.001*			
Easterly	0.417	0.003	0.007	0.001*		
Kempner	0.055	0.450	0.017	0.001*	0.031	
Upper	0.001*	0.010	0.004	0.001*	0.001*	0.001*
Two Weeks	Academy	Bandera	Comfort	Driftwood	Easterly	Kempner
Bandera	0.004					
Comfort	0.020	0.259				
Driftwood	0.001*	0.001*	0.004			
Easterly	0.79	0.013	0.048	0.001*		
Kempner	0.001*	0.375	0.246	0.001*	0.002*	
Upper	0.003	0.001*	0.005	0.021	0.004	0.001*
Four Weeks	Academy	Bandera	Comfort	Driftwood	Easterly	Kempner
Bandera	0.009					
Comfort	0.023	0.638				
Driftwood	0.001*	0.001*	0.002*			
Easterly	0.642	0.079	0.044	0.007		
Kempner	0.034	0.804	0.554	0.001*	0.106	
Upper	0.001*	0.001*	0.001*	0.001*	0.001*	0.001*
Six Weeks	Academy	Bandera	Comfort	Driftwood	Easterly	Kempner
Bandera	0.021					
Comfort	0.129	0.593				
Driftwood	0.006	0.001*	0.001*			
Easterly	0.797	0.087	0.158	0.013		
Kempner	0.069	0.510	0.371	0.001*	0.099	
Upper	0.001*	0.001*	0.001*	0.001*	0.002*	0.001*
Eight Weeks	Academy	Bandera	Comfort	Driftwood	Easterly	Kempner
Bandera	0.011					
Comfort	0.068	0.615				
Driftwood	0.002*	0.001*	0.001*			
Easterly	0.598	0.017	0.087	0.009		
Kempner	0.018	0.794	0.387	0.001*	0.026	
Upper	0.001*	0.001*	0.004	0.001*	0.002*	0.001*

Pairwise Procrustes ANOVA comparison P-values for *C*. *venusta* by site within each time period, from field to eight weeks.

*indicates a significant difference after Bonferroni correction.

## Discussion

These findings support that preservation alters the body shape of fishes across multiple taxa, between populations, and through time in varying ways. We found significant differences in morphology across preservation time in five of the 10 species examined, significant shrinkage in centroid size, and differences in body shape by collection locale across time periods. Our results were similar to previous geomorphometric preservation studies in that morphology changed with preservation [18,20]. However, results from this study provide a more complete understanding of specific morphometric changes that occur over preservation time across a greater taxonomic range. We also assessed changes in both the formalin and ethanol stages of preservation, as our fishes were stored in formalin for two weeks, then subsequently stored in ethanol. Our results are consistent with previous research which found that formalin preservation tends to decrease the overall size of specimens [3,6,7]. These results similarly find decreases in overall sizes of individuals, yet each body area responds to the treatment differently. In general, the centroid size of specimens was smaller at 2W than in field, indicating an overall decrease in specimen sizes during formalin preservation. However, there was an increase in body area in the head and caudal areas and shrinkage in other areas of the body, which indicates that formalin preservation introduces variation which can be difficult to discern.

Previous work has shown that fishes tend to shrink when preserved in ethanol [3–6]. Previous studies, which assessed short-term (i.e. < 1 year) changes in geometric morphology due to preservation in ethanol, found that the largest changes occurred in the eye and body depth or body size post-preservation when compared to fresh specimens [18,20], whereas long-term changes (i.e. > 10 years) were not significant for preserved specimens only [24]. In this study, there was an expansion of the abdominal area in 2W and 4W with no change or shrinking in 6W and 8W. Additionally, from field to 8W there was a trend of increased body depth and decreased abdomen length. Changes to the eye character was present, but minor in both the shape change plots and in the PC loadings. This study enhanced resolution and expanded scope by addressing changes to various body region areas over time in multiple species.

The varying changes that occur at different stages in the time periods could be related to several factors. Changes from field to 2W (formalin fixation period) may indicate the formalin stage of preservation tends to expand muscular body areas such as the caudal region and pectoral girdle, yet after subsequent ethanol preservation, these areas may become dehydrated and decrease in size [43], although this did not occur consistently in all fishes in this study. The muscle and visceral organization of each species, including swim bladder presence (e.g., Cyprinidae) or absence (e.g., Percidae), may be differentially affected by formalin-ethanol preservation and could lead to inconsistent measurements that were observed across preservation time periods in our morphometric analyses. Additionally, high variability in water retention in the muscle tissue of species could affect morphological changes during the preservation process [5].

There are potential sources of error in this study, which apply to geometric morphometric studies in general. Some of the trends seen in changes in body shape could be due to a jar effect, where storage influenced specimens’ body shape, potentially causing them to arch [24]. However, we attempted to alleviate this issue by storing each fish in individual vials and pinning specimens while photographing them. On several species, the first principal component axis was predominately associated with body arching. Previous work has found that random body posture differences can cause body arching to be an issue with morphological studies and strongly associate with the first principal component axis [44]. However, specimens become more rigid and difficult to pin into place after preservation [43], likely due to a combination of a jar effect and the preservation process, thus potentially increasing differences related to random body postures in subsequent time periods. Another potential complicating factor of this study deals with measurement error. While past studies have found that measurement error is a comparatively small component of total variance of the data [36], we did try to minimize the effect of measurement error in our study design while not directly testing for it. Standardized data acquisition procedures, including having a single individual place landmarks on all specimens of the same species for all time periods, using a camera mount that ensured a constant focal length and specimen placement, and removal of specimen outliers were implemented in order to mitigate error. Lastly, smaller sample sizes (e.g. *P*. *carbonaria*) could have an impact on our analyses, however, the results are mostly consistent with other species which had larger sample sizes.

Many studies have performed geometric morphometric analyses using preserved fishes in a variety of biological, ecological, or evolutionary contexts [10,13,14,21–23]. These studies attributed divergent body shapes to various environmental or ecological factors, even when using preserved specimens [10,13,14,21–23]. In this study, morphological changes in *C*. *lutrensis* and *C*. *venusta* occurred throughout the preservation time periods based on their collection sites over the eight weeks, which could influence conclusions derived from these data. If the field time period was examined, one would have come to different conclusions than if one were to have used photographs from any of the preserved time periods. For example, at the field time period of *C*. *lutrensis*, one could have concluded that Cuero and Gonzalez possess significantly different body shapes. In contrast, if one used the specimens after two weeks of preservation, we would have concluded they were not significantly different morphologically. Additionally, the magnitude of changes differed between pairwise site comparisons and time periods relative to field. The largest absolute percent change in Procrustes distances occurred at 6W relative to field and the wide standard deviations indicate a high amount of variability in body shape. These changes in body shape were due to the preservation process because the same individuals from the same populations were used and compared over the different time periods.

Overall this study demonstrates fish body shapes vary within the same species over preservation time, across different species, and within the same species by collection locale. Future studies comparing differences in body shapes should be aware of the potential effects that preservation has on morphology across different taxonomic levels and collection locales and take caution when performing morphometrics comparing fresh and/or preserved specimens. If possible, we would recommend the use of fresh specimens. Since this is not always possible, depending on the taxa being studied, setting aside a subset of specimens and assessing changes after preservation relative to fresh field could be performed [45] or if body arching is an issue, those individuals could be removed from the analyses [23]. Here, we show that body shapes can change in inconsistent, varying, and complicating ways after preservation.

## Supporting information

S1 Table Sample sizes for *C*. *lutrensis* and *C*. *venusta* for each site. Sample sizes for *C*. *lutrensis* and *C*. *venusta* for each sampling site used in pairwise site comparisons.
(DOCX)

S2 Table Generalized linear models testing centroid size estimates by time for cyprinids and poeciliids. Results from the generalized linear models testing centroid size estimates by time periods for all cyprinids and poeciliids. Included are the slope estimate, standard error, degrees of freedom, t-value, p-value, and centroid size estimate from the model. Field represents measurements taken on freshly dead specimens, followed by preservation of two weeks (2W) through eight weeks (8W).
(DOCX)

S3 Table Generalized linear models testing centroid size estimates by time for percids. Results from the generalized linear models testing centroid size estimates by time periods for all percids. Included are the slope estimate, standard error, degrees of freedom, t-value, p-value, and centroid size estimate from the model. Field represents measurements taken on freshly dead specimens, followed by preservation of two weeks (2W) through eight weeks (8W).
(DOCX)

S4 Table Procrustes distances between sites within each time period for *C*. *lutrensis*. Procrustes distances calculated for each pairwise site comparison for *C*. *lutrensis* within each time period.
(DOCX)

S5 Table Procrustes distances between sites within each time period for *C*. *venusta*. Procrustes distances calculated for each pairwise site comparison for *C*. *venusta* within each time period.
(DOCX)

S6 Table Absolute percent change in Procrustes distances for *C*. *lutrensis*. Absolute percent changes in procrustes distances for each pairwise comparison for *C*. *lutrensis* between field and all subsequent time periods. Included are pairwise percent changes for each pairwise site comparison, the mean, standard deviation, absolute mean, and absolute standard deviation (STDEV) for each time period comparison.
(DOCX)

S7 Table Absolute percent change in Procrustes distances for *C*. *venusta*. Absolute percent changes in procrustes distances for each pairwise comparison for *C*. *venusta* between field and all subsequent time periods. Included are pairwise percent changes for each pairwise site comparison, the mean, standard deviation, absolute mean, and absolute standard deviation (STDEV) for each time period comparison.
(DOCX)

S1 Fig Plots of PC axes 1 and 2 for each species. Mean of each principle components axis 1 and 2 for each species: A) *C*. *venusta*, B) *G*. *geiseri*, C) *C*. *lutrensis*, D) *M*. *marconis*, E) *N*. *amabilis*, F) *N*. *chalybaeus*, G) *P*. *apristis*, H) *P*. *carbonaria*, I) *E*. *spectabile*. Error bars represent one standard deviation.
(ZIP)

S2 Fig Plots of PC minimum and maximum shapes. Shape plots of minimum (black lines and points) and maximum (dark gray lines and points) PC axis values for PC 1–5 for each species. A) *C*. *venusta*, B) *G*. *geiseri*, C) *M*. *hyostoma*, D) *C*. *lutrensis*, E) *M*. *marconis*, F) *N*. *amabilis*, G) *N*. *chalybaeus*, H) *P*. *apristis*, I) *P*. *carbonaria*, J) *E*. *spectabile*.
(ZIP)

S3 Fig Trajectory analysis for each species of each time period relative to field. Trajectory analysis of time period relative to field for each species; shows mean shape at a particular time period (2W, 4W, 6W, and 8W) of preservation. A) *C*. *venusta*, B) *G*. *geiseri*, C) *C*. *lutrensis*, D) *M*. *marconis*, E) *N*. *amabilis*, F) *N*. *chalybaeus*, G) *P*. *apristis*, H) *P*. *carbonaria*, I) *E*. *spectabile*.
(ZIP)

S1 Dataset EffectsOfPreservation_Data.zip contains the data for this study including TPS files containing landmark coordinates and classifier information (e.g. time period and individual identifier) for each species. Included additionally for *C*. *lutrensis* and *C*. *venusta* are river and site of capture.
(ZIP)
