# Supplementary material for: Effect of preservation on fish morphology over time: Implications for morphological studies
Source: PLoS One. 2019 Mar 21;14(3):e0213915. doi: 10.1371/journal.pone.0213915 (PMC6428252; doi:10.1371/journal.pone.0213915)
Supplement: S1 Table — Sample sizes for C. lutrensis and C. venusta for each sampling site used in pairwise site comparisons. (DOCX) [file pone.0213915.s001.docx]

S1 Table. **Sample sizes for *C. lutrensis* and *C. venusta* for each site.** Sample sizes for *C. lutrensis* and *C. venusta* for each sampling site used in pairwise site comparisons.

| Species | Site | N |
| --- | --- | --- |
| *C. lutrensis* | Academy | 10 |
|  | Cuero | 7 |
|  | Goliad | 5 |
|  | Gonzalez | 12 |
| *C. venusta* | Academy | 8 |
|  | Bendera | 9 |
|  | Comfort | 10 |
|  | Driftwood | 17 |
|  | Easterly | 8 |
|  | Kempner | 10 |
|  | Upper | 12 |
