# Supplementary material for: Effect of preservation on fish morphology over time: Implications for morphological studies
Source: PLoS One. 2019 Mar 21;14(3):e0213915. doi: 10.1371/journal.pone.0213915 (PMC6428252; doi:10.1371/journal.pone.0213915)
Supplement: S2 Table — Results from the generalized linear models testing centroid size estimates by time periods for all cyprinids and poeciliids. Included are the slope estimate, standard error, degrees of freedom, t-value, p-value, and centroid size estimate from the model. Field represents measurements taken on freshly dead specimens, followed by preservation of two weeks (2W) through eight weeks (8W). (DOCX) [file pone.0213915.s002.docx]

Table S2. **Generalized linear models testing centroid size estimates by time for cyprinids and poeciliids.** Results from the generalized linear models testing centroid size estimates by time periods for all cyprinids and poeciliids. Included are the slope estimate, standard error, degrees of freedom, t-value, p-value, and centroid size estimate from the model. Field represents measurements taken on freshly dead specimens, followed by preservation of two weeks (2W) through eight weeks (8W).

| Species | Time | Slope Estimate | Standard Error | Degrees of Freedom | T-value | P-value | Centroid Size Estimate |
| --- | --- | --- | --- | --- | --- | --- | --- |
| *C. lutrensis* | Field | 56.4088 | 1.0654 | 144 | 52.95 | <0.0001 | 56.41 |
|  | 2W | -0.8038 | 0.3588 | 144 | -2.24 | 0.0266 | 55.60 |
|  | 4W | -2.2898 | 0.3588 | 144 | -6.38 | <0.0001 | 54.12 |
|  | 6W | -3.8681 | 0.3588 | 144 | -10.78 | <0.0001 | 52.54 |
|  | 8W | -3.3500 | 0.3588 | 144 | -9.34 | <0.0001 | 53.06 |
| *C. venusta* | Field | 70.3107 | 1.9487 | 292 | 36.08 | <0.0001 | 70.31 |
|  | 2W | -0.3344 | 0.5189 | 292 | -0.64 | 0.5199 | 69.98 |
|  | 4W | -2.5226 | 0.5189 | 292 | -4.86 | <0.0001 | 67.79 |
|  | 6W | -2.6399 | 0.5189 | 292 | -5.09 | <0.0001 | 67.67 |
|  | 8W | -3.7072 | 0.5189 | 292 | -7.14 | <0.0001 | 66.60 |
| *M. hyostoma* | Field | 37.1044 | 0.7272 | 56 | 51.03 | <0.0001 | 37.10 |
|  | 2W | -0.0294 | 0.1737 | 56 | -0.17 | 0.8660 | 37.07 |
|  | 4W | -1.2546 | 0.1737 | 56 | -7.22 | <0.0001 | 35.85 |
|  | 6W | -0.3128 | 0.1737 | 56 | -1.80 | 0.0771 | 36.79 |
|  | 8W | -1.1360 | 0.1737 | 56 | -6.54 | <0.0001 | 35.97 |
| *M. marconis* | Field | 51.6067 | 0.9999 | 152 | 51.61 | <0.0001 | 51.61 |
|  | 2W | -1.0674 | 0.3211 | 152 | -3.32 | 0.0011 | 50.54 |
|  | 4W | -1.6327 | 0.3211 | 152 | -5.08 | <0.0001 | 49.97 |
|  | 6W | -3.2068 | 0.3211 | 152 | -9.99 | <0.0001 | 48.40 |
|  | 8W | -2.4459 | 0.3211 | 152 | -7.62 | <0.0001 | 49.16 |
| *N. amabilis* | Field | 60.4996 | 1.1298 | 52 | 53.55 | <0.0001 | 60.50 |
|  | 2W | -0.3482 | 0.1877 | 52 | -1.85 | 0.0693 | 60.15 |
|  | 4W | -1.2855 | 0.1877 | 52 | -6.85 | <0.0001 | 59.21 |
|  | 6W | -2.1392 | 0.1877 | 52 | -11.40 | <0.0001 | 58.36 |
|  | 8W | -2.2619 | 0.1877 | 52 | -12.05 | <0.0001 | 58.24 |
| *N. chalybaeus* | Field | 52.3431 | 0.5592 | 20 | 93.61 | <0.0001 | 52.34 |
|  | 2W | -0.7714 | 0.4367 | 20 | -1.77 | 0.0926 | 51.57 |
|  | 4W | -2.4210 | 0.4367 | 20 | -5.54 | <0.0001 | 49.92 |
|  | 6W | -4.0875 | 0.4367 | 20 | -9.36 | <0.0001 | 48.26 |
|  | 8W | -3.6421 | 0.4367 | 20 | -8.34 | <0.0001 | 48.70 |
| *G. geiseri* | Field | 38.9661 | 0.5146 | 80 | 75.73 | <0.0001 | 38.97 |
|  | 2W | -0.1543 | 0.1158 | 80 | -1.33 | 0.1864 | 38.81 |
|  | 4W | -0.7380 | 0.1158 | 80 | -6.37 | <0.0001 | 38.23 |
|  | 6W | -1.4577 | 0.1158 | 80 | -12.59 | <0.0001 | 37.51 |
|  | 8W | -1.3547 | 0.1158 | 80 | -11.70 | <0.0001 | 37.61 |
