# Supplementary material for: Effect of preservation on fish morphology over time: Implications for morphological studies
Source: PLoS One. 2019 Mar 21;14(3):e0213915. doi: 10.1371/journal.pone.0213915 (PMC6428252; doi:10.1371/journal.pone.0213915)
Supplement: S3 Table — Results from the generalized linear models testing centroid size estimates by time periods for all percids. Included are the slope estimate, standard error, degrees of freedom, t-value, p-value, and centroid size estimate from the model. Field represents measurements taken on freshly dead specimens, followed by preservation of two weeks (2W) through eight weeks (8W). (DOCX) [file pone.0213915.s003.docx]

Table S3. **Generalized linear models testing centroid size estimates by time for percids**. Results from the generalized linear models testing centroid size estimates by time periods for all percids. Included are the slope estimate, standard error, degrees of freedom, t-value, p-value, and centroid size estimate from the model. Field represents measurements taken on freshly dead specimens, followed by preservation of two weeks (2W) through eight weeks (8W).

| Species | Time | Slope Estimate | Standard Error | Degrees of Freedom | T-value | P-value | Centroid Size Estimate |
| --- | --- | --- | --- | --- | --- | --- | --- |
| *E. spectabile* | Field | 48.3846 | 0.9621 | 68 | 50.29 | <0.0001 | 48.38 |
|  | 2W | -1.0865 | 0.4879 | 68 | -2.23 | 0.0293 | 47.30 |
|  | 4W | -1.5289 | 0.4879 | 68 | -3.13 | 0.0025 | 46.86 |
|  | 6W | -1.1651 | 0.4879 | 68 | -2.39 | 0.0197 | 47.22 |
|  | 8W | -1.6108 | 0.4879 | 68 | -3.30 | 0.0015 | 46.77 |
| *P. apristis* | Field | 78.3520 | 3.4610 | 44 | 22.64 | <0.0001 | 78.35 |
|  | 2W | 0.2691 | 0.4551 | 44 | 0.59 | 0.5573 | 78.62 |
|  | 4W | 0.2498 | 0.4551 | 44 | 0.55 | 0.5858 | 78.60 |
|  | 6W | -1.4068 | 0.4551 | 44 | -3.09 | 0.0035 | 76.95 |
|  | 8W | -2.2606 | 0.4551 | 44 | -4.97 | <0.0001 | 76.09 |
| *P. carbonaria* | Field | 115.4973 | 5.6899 | 20 | 20.30 | <0.0001 | 115.50 |
|  | 2W | -2.1430 | 2.9677 | 20 | -0.72 | 0.4786 | 113.35 |
|  | 4W | 2.0125 | 2.9677 | 20 | 0.68 | 0.5055 | 117.51 |
|  | 6W | 3.3412 | 2.9677 | 20 | 1.13 | 0.2736 | 118.84 |
|  | 8W | 2.3294 | 2.9677 | 20 | 0.78 | 0.4417 | 117.83 |
