# Supplementary material for: Effect of preservation on fish morphology over time: Implications for morphological studies
Source: PLoS One. 2019 Mar 21;14(3):e0213915. doi: 10.1371/journal.pone.0213915 (PMC6428252; doi:10.1371/journal.pone.0213915)
Supplement: S4 Table — Procrustes distances calculated for each pairwise site comparison for C. lutrensis within each time period. (DOCX) [file pone.0213915.s004.docx]

S4 Table. **Procrustes distances between sites within each time period for *C. lutrensis.*** Procrustes distances calculated for each pairwise site comparison for *C. lutrensis* within each time period.

| Field | Academy | Cuero | Goliad |
| --- | --- | --- | --- |
| Cuero | 0.018 |  |  |
| Goliad | 0.037 | 0.041 |  |
| Gonzalez | 0.026 | 0.027 | 0.036 |
|  |  |  |  |
| Two Weeks | Academy | Cuero | Goliad |
| Cuero | 0.026 |  |  |
| Goliad | 0.033 | 0.029 |  |
| Gonzalez | 0.022 | 0.016 | 0.022 |
|  |  |  |  |
| Four Weeks | Academy | Cuero | Goliad |
| Cuero | 0.030 |  |  |
| Goliad | 0.042 | 0.025 |  |
| Gonzalez | 0.026 | 0.014 | 0.024 |
|  |  |  |  |
| Six Weeks | Academy | Cuero | Goliad |
| Cuero | 0.038 |  |  |
| Goliad | 0.044 | 0.026 |  |
| Gonzalez | 0.031 | 0.014 | 0.025 |
|  |  |  |  |
| Eight Weeks | Academy | Cuero | Goliad |
| Cuero | 0.031 |  |  |
| Goliad | 0.035 | 0.036 |  |
| Gonzalez | 0.023 | 0.015 | 0.031 |
