# Supplementary material for: Effect of preservation on fish morphology over time: Implications for morphological studies
Source: PLoS One. 2019 Mar 21;14(3):e0213915. doi: 10.1371/journal.pone.0213915 (PMC6428252; doi:10.1371/journal.pone.0213915)
Supplement: S5 Table — Procrustes distances calculated for each pairwise site comparison for C. venusta within each time period. (DOCX) [file pone.0213915.s005.docx]

S5 Table. **Procrustes distances between sites within each time period for *C. venusta.*** Procrustes distances calculated for each pairwise site comparison for *C. venusta* within each time period.

| Field | Academy | Bendera | Comfort | Driftwood | Easterly | Kempner |
| --- | --- | --- | --- | --- | --- | --- |
| Bendera | 0.027 |  |  |  |  |  |
| Comfort | 0.031 | 0.017 |  |  |  |  |
| Driftwood | 0.033 | 0.028 | 0.030 |  |  |  |
| Easterly | 0.017 | 0.028 | 0.027 | 0.038 |  |  |
| Kempner | 0.022 | 0.015 | 0.024 | 0.028 | 0.023 |  |
| Upper | 0.034 | 0.024 | 0.027 | 0.031 | 0.038 | 0.032 |
|  |  |  |  |  |  |  |
| Two Weeks | Academy | Bendera | Comfort | Driftwood | Easterly | Kempner |
| Bendera | 0.034 |  |  |  |  |  |
| Comfort | 0.027 | 0.018 |  |  |  |  |
| Driftwood | 0.032 | 0.031 | 0.026 |  |  |  |
| Easterly | 0.013 | 0.028 | 0.023 | 0.030 |  |  |
| Kempner | 0.036 | 0.017 | 0.018 | 0.036 | 0.032 |  |
| Upper | 0.031 | 0.029 | 0.029 | 0.021 | 0.028 | 0.039 |
|  |  |  |  |  |  |  |
| Four Weeks | Academy | Bendera | Comfort | Driftwood | Easterly | Kempner |
| Bendera | 0.030 |  |  |  |  |  |
| Comfort | 0.026 | 0.013 |  |  |  |  |
| Driftwood | 0.029 | 0.032 | 0.029 |  |  |  |
| Easterly | 0.014 | 0.022 | 0.023 | 0.025 |  |  |
| Kempner | 0.025 | 0.012 | 0.014 | 0.030 | 0.020 |  |
| Upper | 0.038 | 0.030 | 0.032 | 0.031 | 0.034 | 0.034 |
|  |  |  |  |  |  |  |
| Six Weeks | Academy | Bendera | Comfort | Driftwood | Easterly | Kempner |
| Bendera | 0.027 |  |  |  |  |  |
| Comfort | 0.021 | 0.014 |  |  |  |  |
| Driftwood | 0.026 | 0.035 | 0.032 |  |  |  |
| Easterly | 0.013 | 0.023 | 0.020 | 0.024 |  |  |
| Kempner | 0.024 | 0.015 | 0.016 | 0.037 | 0.021 |  |
| Upper | 0.041 | 0.042 | 0.037 | 0.036 | 0.033 | 0.042 |
|  |  |  |  |  |  |  |
| Eight Weeks | Academy | Bendera | Comfort | Driftwood | Easterly | Kempner |
| Bendera | 0.030 |  |  |  |  |  |
| Comfort | 0.022 | 0.013 |  |  |  |  |
| Driftwood | 0.028 | 0.034 | 0.030 |  |  |  |
| Easterly | 0.014 | 0.026 | 0.021 | 0.025 |  |  |
| Kempner | 0.027 | 0.011 | 0.015 | 0.037 | 0.024 |  |
| Upper | 0.036 | 0.031 | 0.028 | 0.034 | 0.029 | 0.034 |
