# Supplementary material for: Effect of preservation on fish morphology over time: Implications for morphological studies
Source: PLoS One. 2019 Mar 21;14(3):e0213915. doi: 10.1371/journal.pone.0213915 (PMC6428252; doi:10.1371/journal.pone.0213915)
Supplement: S7 Table — Absolute percent changes in procrustes distances for each pairwise comparison for C. venusta between field and all subsequent time periods. Included are pairwise percent changes for each pairwise site comparison, the mean, standard deviation, absolute mean, and absolute standard deviation (STDEV) for each time period comparison. (DOCX) [file pone.0213915.s007.docx]

| Site Comparison | Field-2W | Field-4W | Field-6W | Field-8W |
| --- | --- | --- | --- | --- |
| Academy-Bendera | 25.926 | 11.111 | 0.000 | 11.111 |
| Academy-Comfort | -12.903 | -16.129 | -32.258 | -29.032 |
| Academy-Driftwood | -3.030 | -12.121 | -21.212 | -15.152 |
| Academy-Easterly | -23.529 | -17.647 | -23.529 | -17.647 |
| Academy-Kempner | 63.636 | 13.636 | 9.091 | 22.727 |
| Academy-Upper | -8.824 | 11.765 | 20.588 | 5.882 |
| Bendera-Comfort | 5.882 | -23.529 | -17.647 | -23.529 |
| Bendera-Driftwood | 10.714 | 14.286 | 25.000 | 21.429 |
| Bendera-Easterly | 0.000 | -21.429 | -17.857 | -7.143 |
| Bendera-Kempner | 13.333 | -20.000 | 0.000 | -26.667 |
| Bendera-Upper | 20.833 | 25.000 | 75.000 | 29.167 |
| Comfort-Driftwood | -13.333 | -3.333 | 6.667 | 0.000 |
| Comfort-Easterly | -14.815 | -14.815 | -25.926 | -22.222 |
| Comfort-Kempner | -25.000 | -41.667 | -33.333 | -37.500 |
| Comfort-Upper | 7.407 | 18.519 | 37.037 | 3.704 |
| Driftwood-Easterly | -21.053 | -34.211 | -36.842 | -34.211 |
| Driftwood-Kempner | 28.571 | 7.143 | 32.143 | 32.143 |
| Driftwood-Upper | -32.258 | 0.000 | 16.129 | 9.677 |
| Easterly-Kempner | 39.130 | -13.043 | -8.696 | 4.348 |
| Easterly-Upper | -26.316 | -10.526 | -13.158 | -23.684 |
| Kempner-Upper | 21.875 | 6.250 | 31.250 | 6.250 |
| Mean | 2.679 | -5.750 | 1.069 | -4.302 |
| Standard Deviation | 24.717 | 18.118 | 28.707 | 21.360 |
| Absolute Mean | 19.922 | 16.008 | 23.017 | 18.249 |
| Absolute STDEV | 14.203 | 9.690 | 16.402 | 11.226 |

**S7 Table. Absolute percent change in Procrustes distances for *C. venusta****.* Absolute percent changes in procrustes distances for each pairwise comparison for *C. venusta* between field and all subsequent time periods. Included are pairwise percent changes for each pairwise site comparison, the mean, standard deviation, absolute mean, and absolute standard deviation (STDEV) for each time period comparison.
