# Supplementary figures and images for: Effect of preservation on fish morphology over time: Implications for morphological studies
Source: PLoS One. 2019 Mar 21;14(3):e0213915. doi: 10.1371/journal.pone.0213915 (PMC6428252; doi:10.1371/journal.pone.0213915)

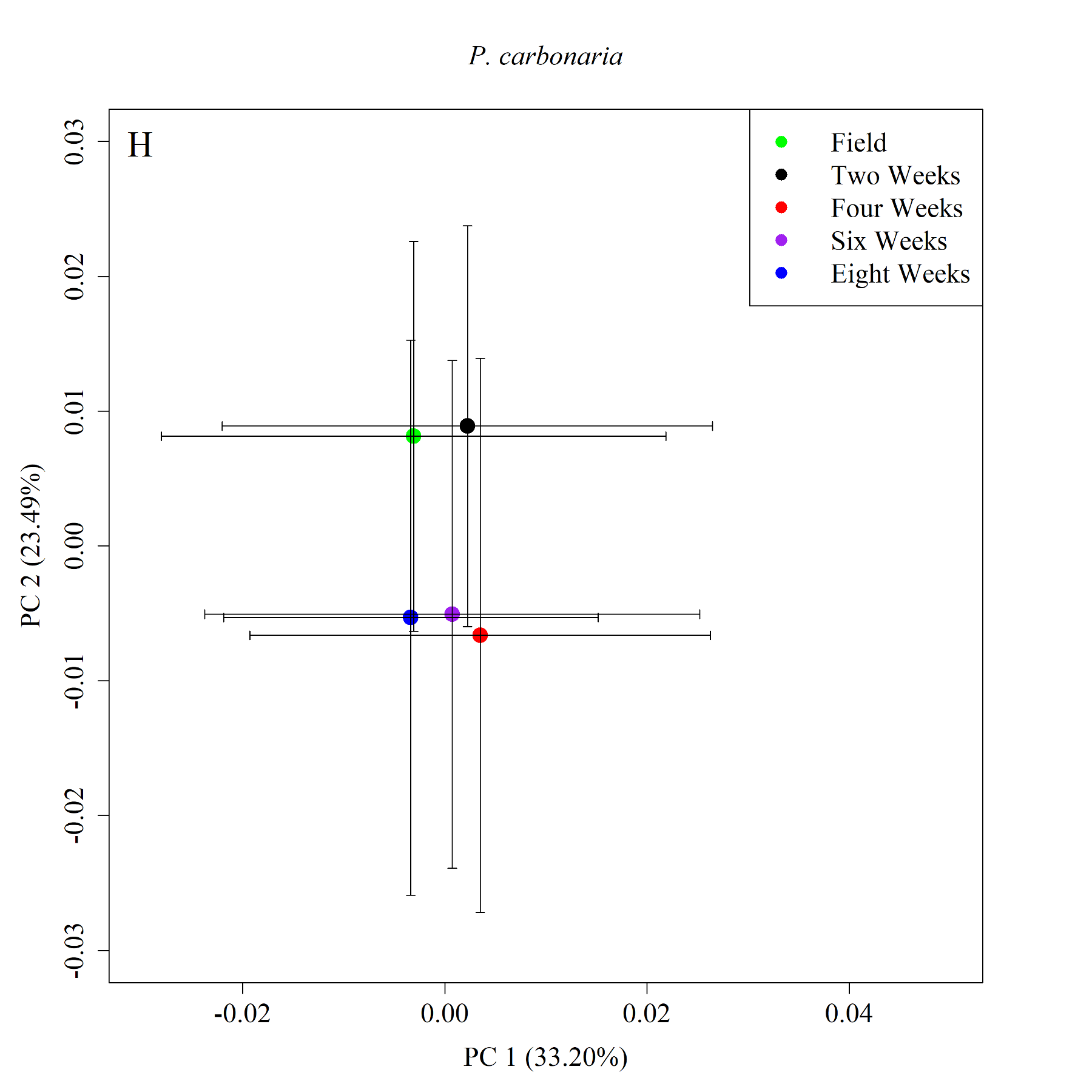

Supplement: S1 Fig — Mean of each principle components axis 1 and 2 for each species: A) C. venusta, B) G. geiseri, C) C. lutrensis, D) M. marconis, E) N. amabilis, F) N. chalybaeus, G) P. apristis, H) P. carbonaria, I) E. spectabile. Error bars represent one standard deviation. (ZIP) [file pone.0213915.s008.zip › Supplemental Fig1h.tif]

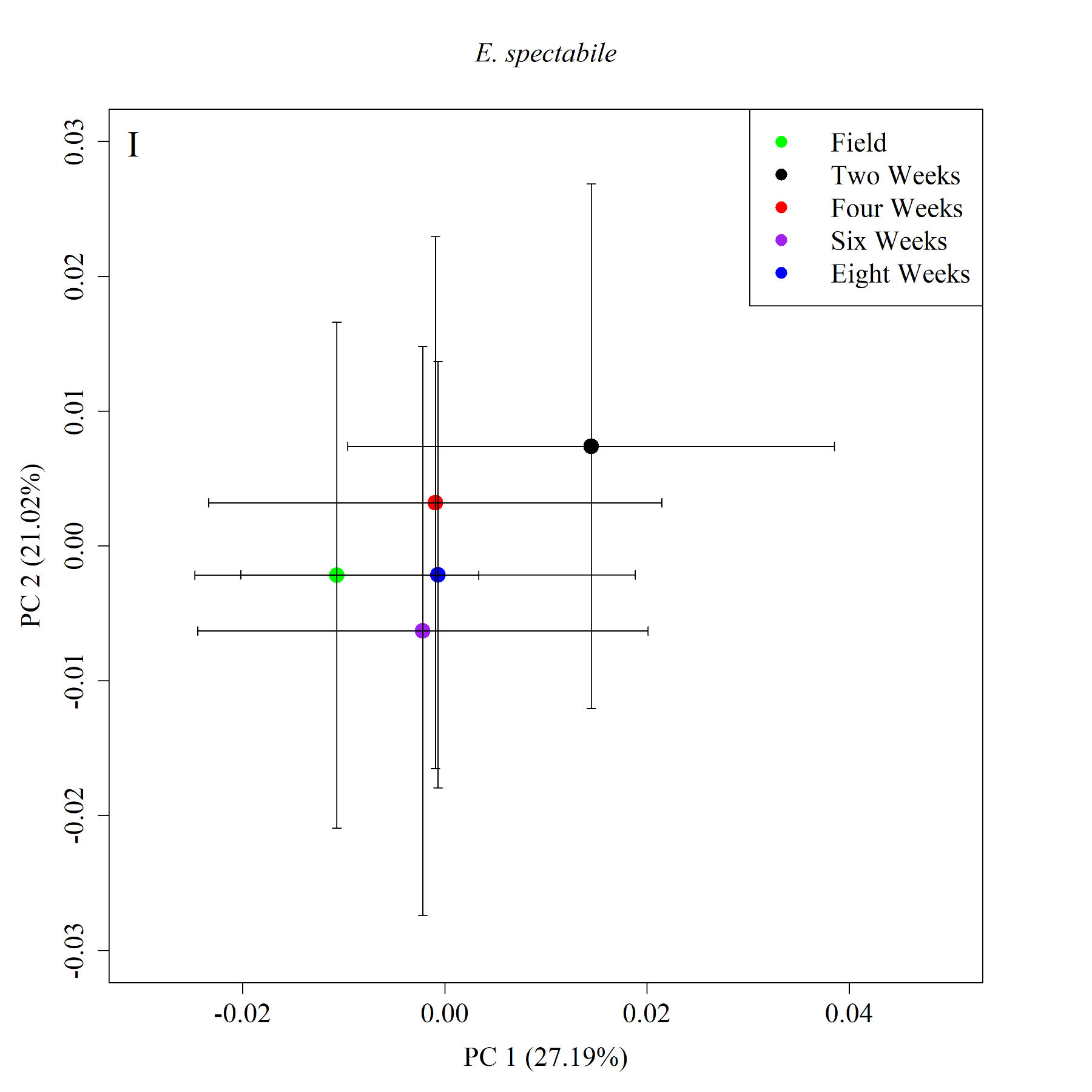

Supplement: S1 Fig — Mean of each principle components axis 1 and 2 for each species: A) C. venusta, B) G. geiseri, C) C. lutrensis, D) M. marconis, E) N. amabilis, F) N. chalybaeus, G) P. apristis, H) P. carbonaria, I) E. spectabile. Error bars represent one standard deviation. (ZIP) [file pone.0213915.s008.zip › Supplemental Fig1i.tif]

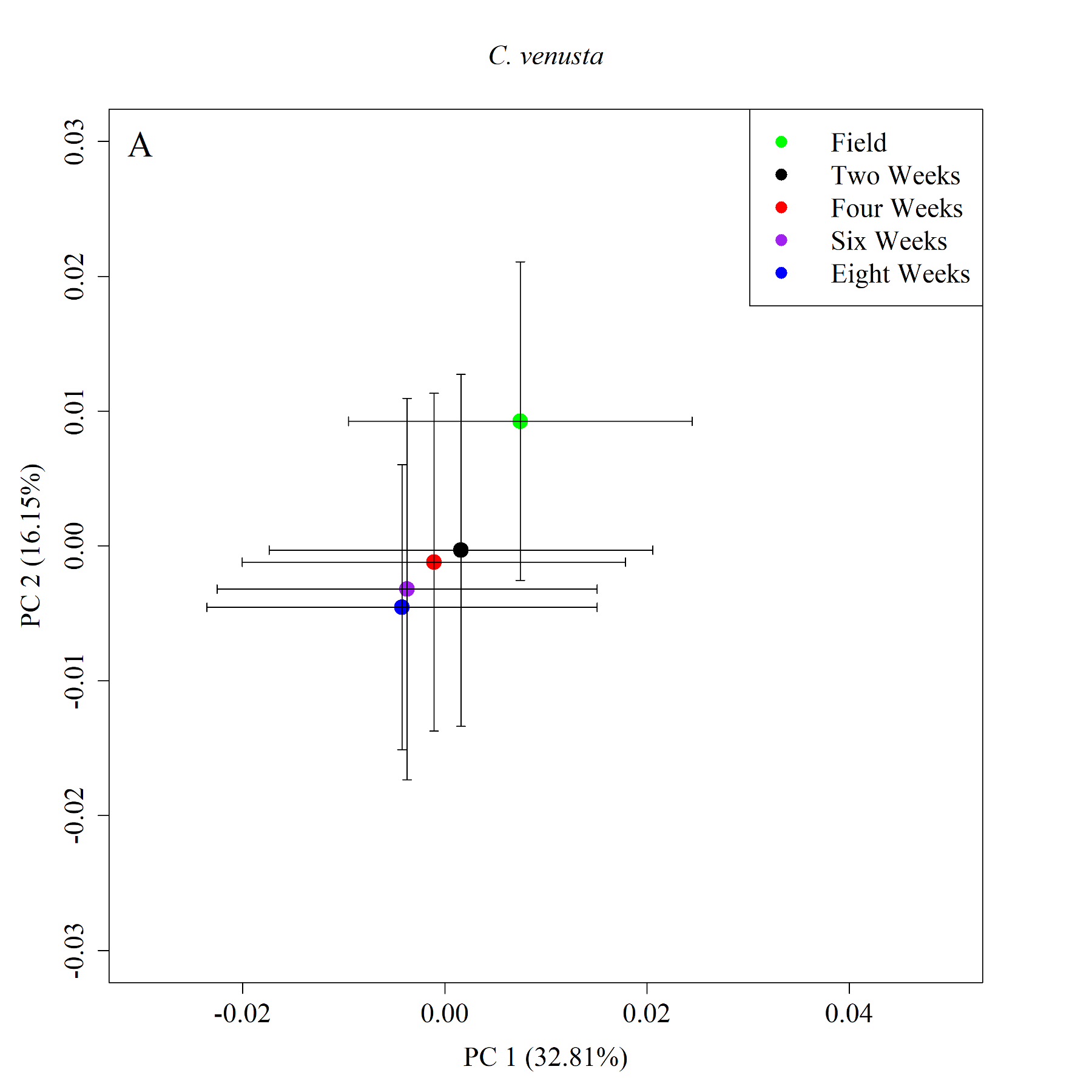

Supplement: S1 Fig — Mean of each principle components axis 1 and 2 for each species: A) C. venusta, B) G. geiseri, C) C. lutrensis, D) M. marconis, E) N. amabilis, F) N. chalybaeus, G) P. apristis, H) P. carbonaria, I) E. spectabile. Error bars represent one standard deviation. (ZIP) [file pone.0213915.s008.zip › Supplemental Fig1a.tif]

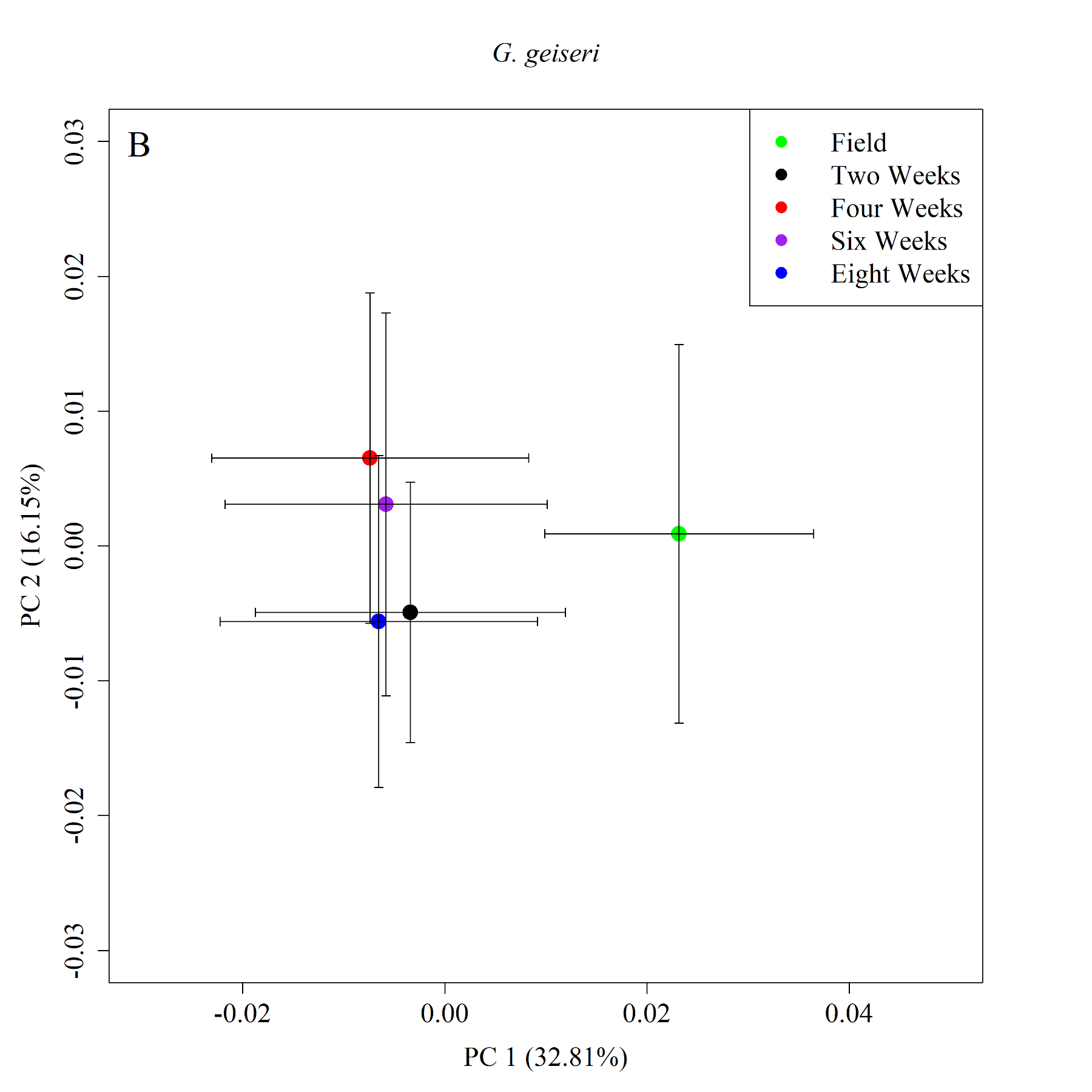

Supplement: S1 Fig — Mean of each principle components axis 1 and 2 for each species: A) C. venusta, B) G. geiseri, C) C. lutrensis, D) M. marconis, E) N. amabilis, F) N. chalybaeus, G) P. apristis, H) P. carbonaria, I) E. spectabile. Error bars represent one standard deviation. (ZIP) [file pone.0213915.s008.zip › Supplemental Fig1b.tif]

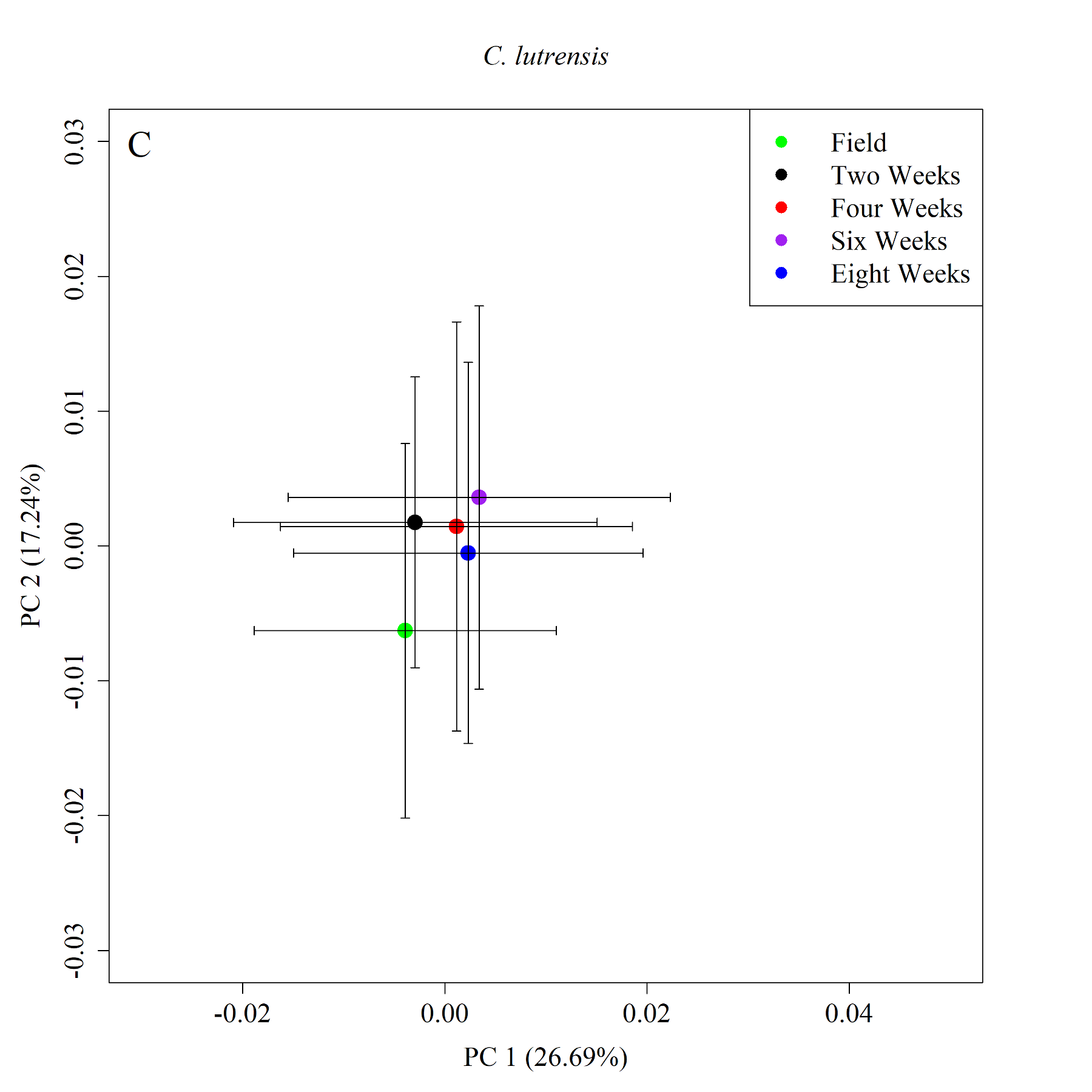

Supplement: S1 Fig — Mean of each principle components axis 1 and 2 for each species: A) C. venusta, B) G. geiseri, C) C. lutrensis, D) M. marconis, E) N. amabilis, F) N. chalybaeus, G) P. apristis, H) P. carbonaria, I) E. spectabile. Error bars represent one standard deviation. (ZIP) [file pone.0213915.s008.zip › Supplemental Fig1c.tif]

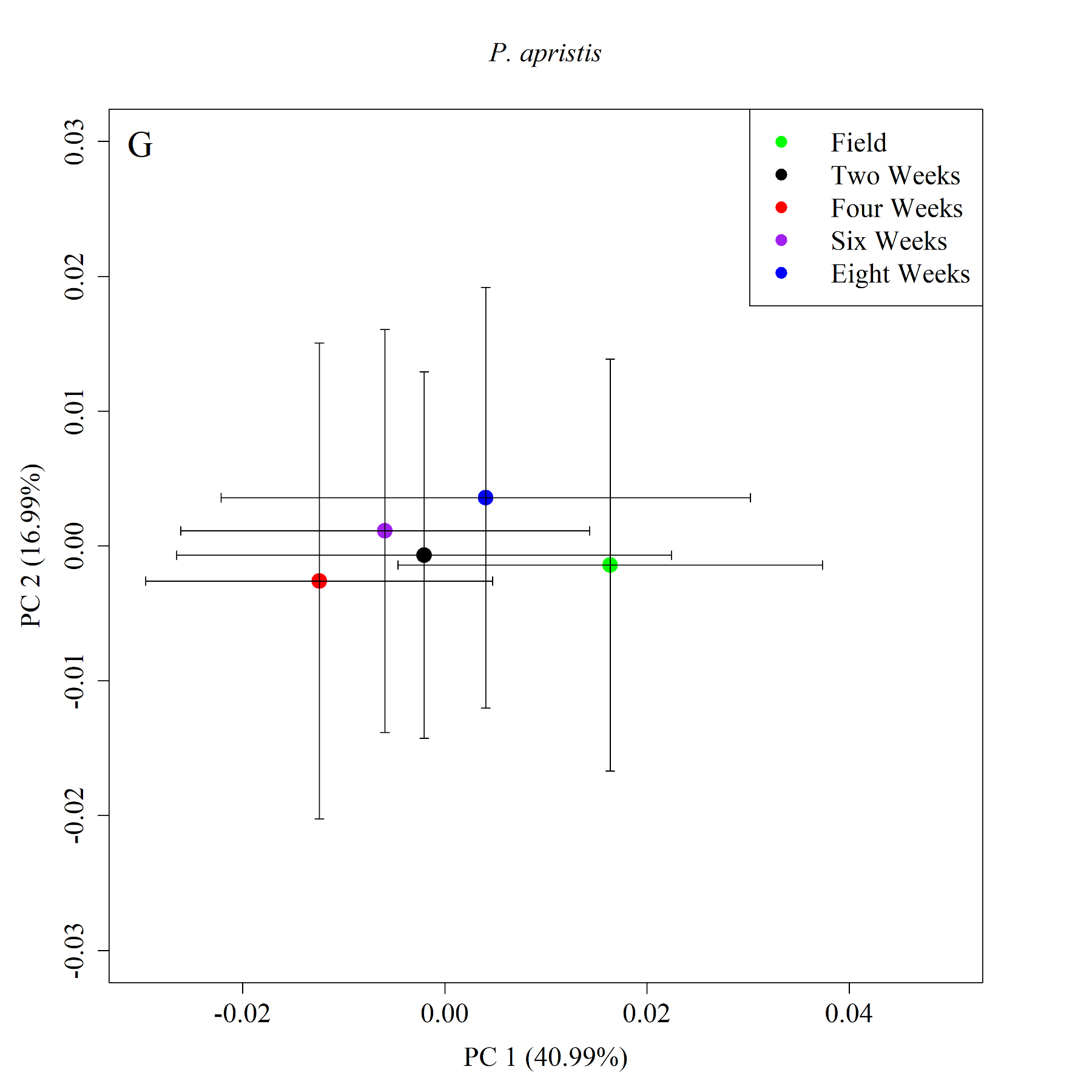

Supplement: S1 Fig — Mean of each principle components axis 1 and 2 for each species: A) C. venusta, B) G. geiseri, C) C. lutrensis, D) M. marconis, E) N. amabilis, F) N. chalybaeus, G) P. apristis, H) P. carbonaria, I) E. spectabile. Error bars represent one standard deviation. (ZIP) [file pone.0213915.s008.zip › Supplemental Fig1g.tif]

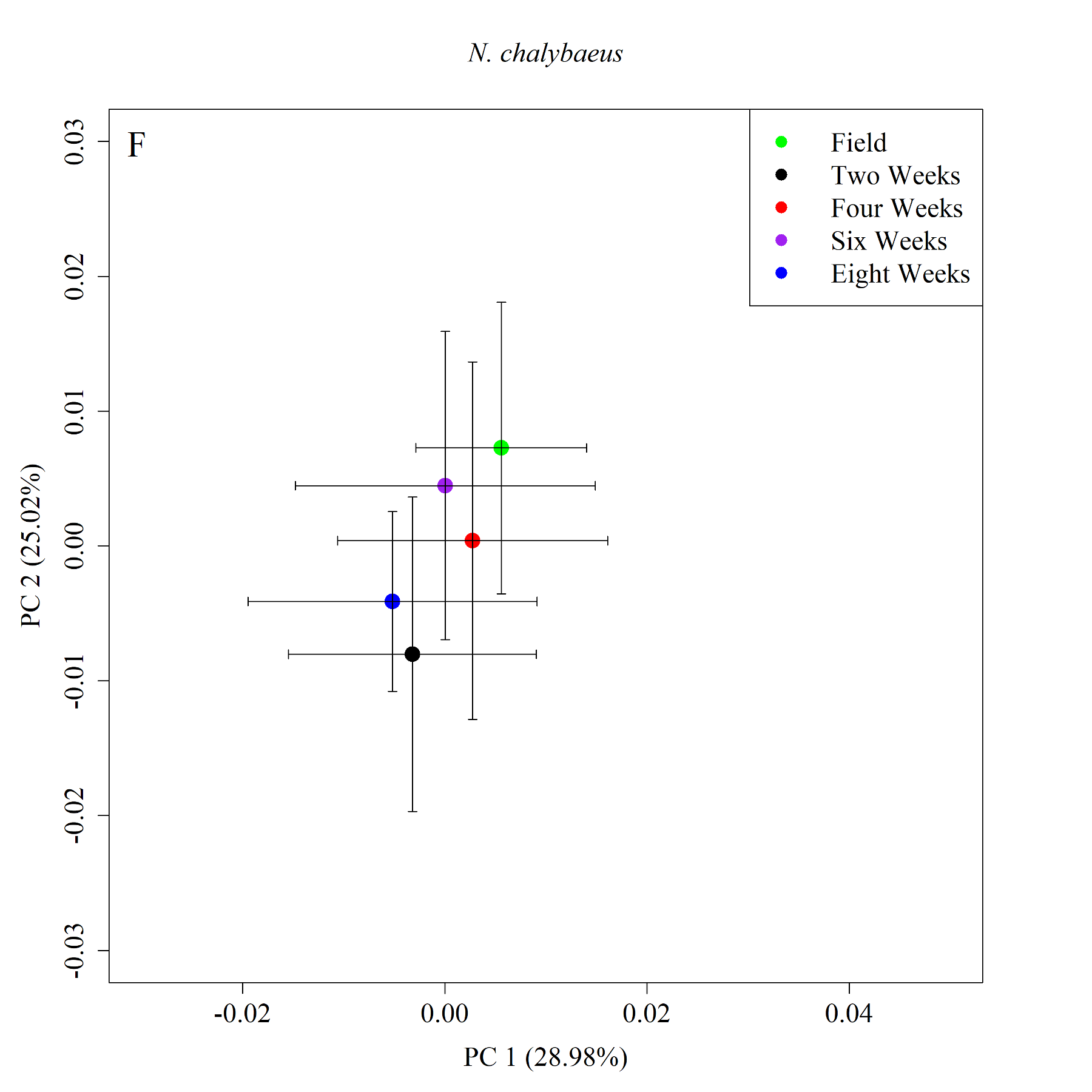

Supplement: S1 Fig — Mean of each principle components axis 1 and 2 for each species: A) C. venusta, B) G. geiseri, C) C. lutrensis, D) M. marconis, E) N. amabilis, F) N. chalybaeus, G) P. apristis, H) P. carbonaria, I) E. spectabile. Error bars represent one standard deviation. (ZIP) [file pone.0213915.s008.zip › Supplemental Fig1f.tif]

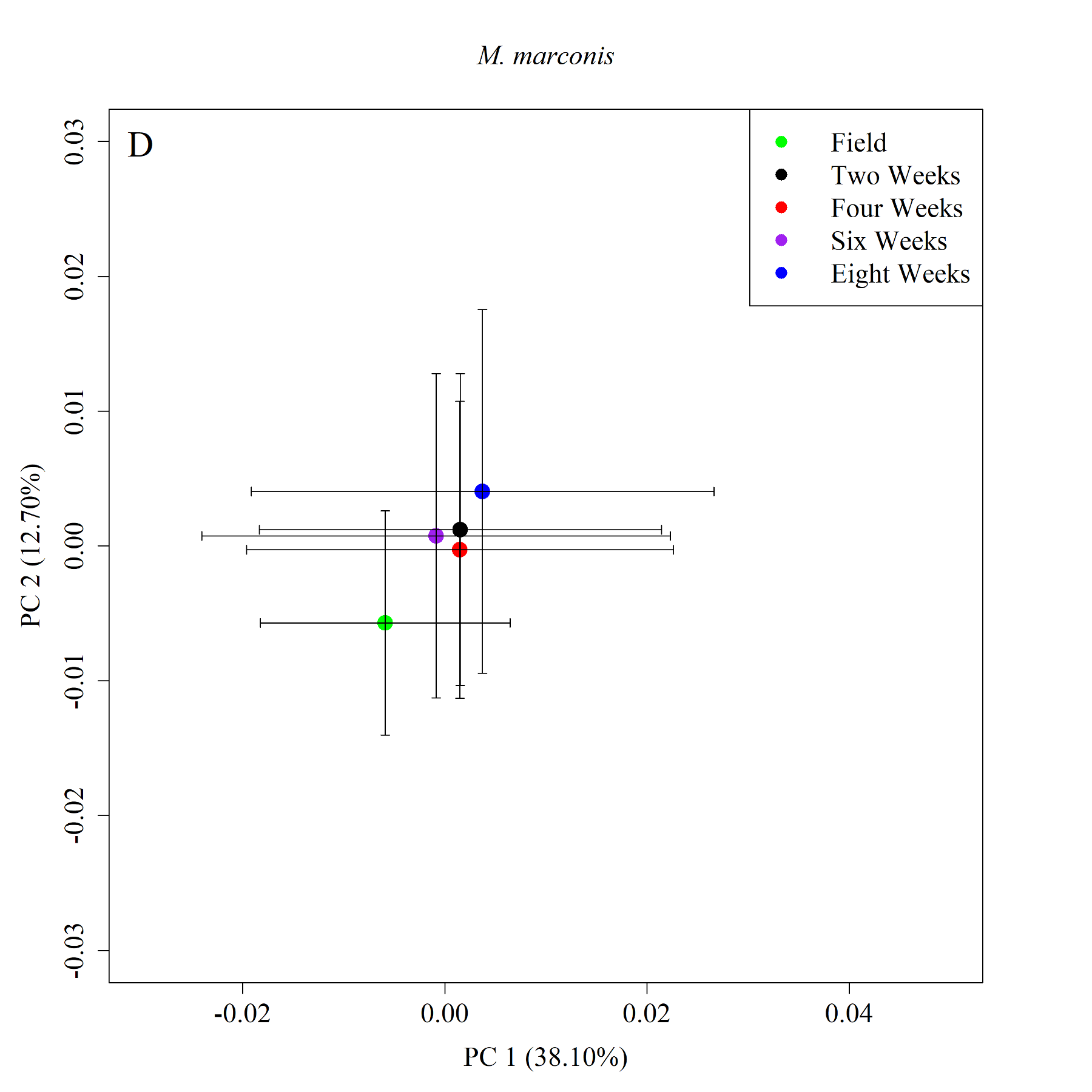

Supplement: S1 Fig — Mean of each principle components axis 1 and 2 for each species: A) C. venusta, B) G. geiseri, C) C. lutrensis, D) M. marconis, E) N. amabilis, F) N. chalybaeus, G) P. apristis, H) P. carbonaria, I) E. spectabile. Error bars represent one standard deviation. (ZIP) [file pone.0213915.s008.zip › Supplemental Fig1d.tif]

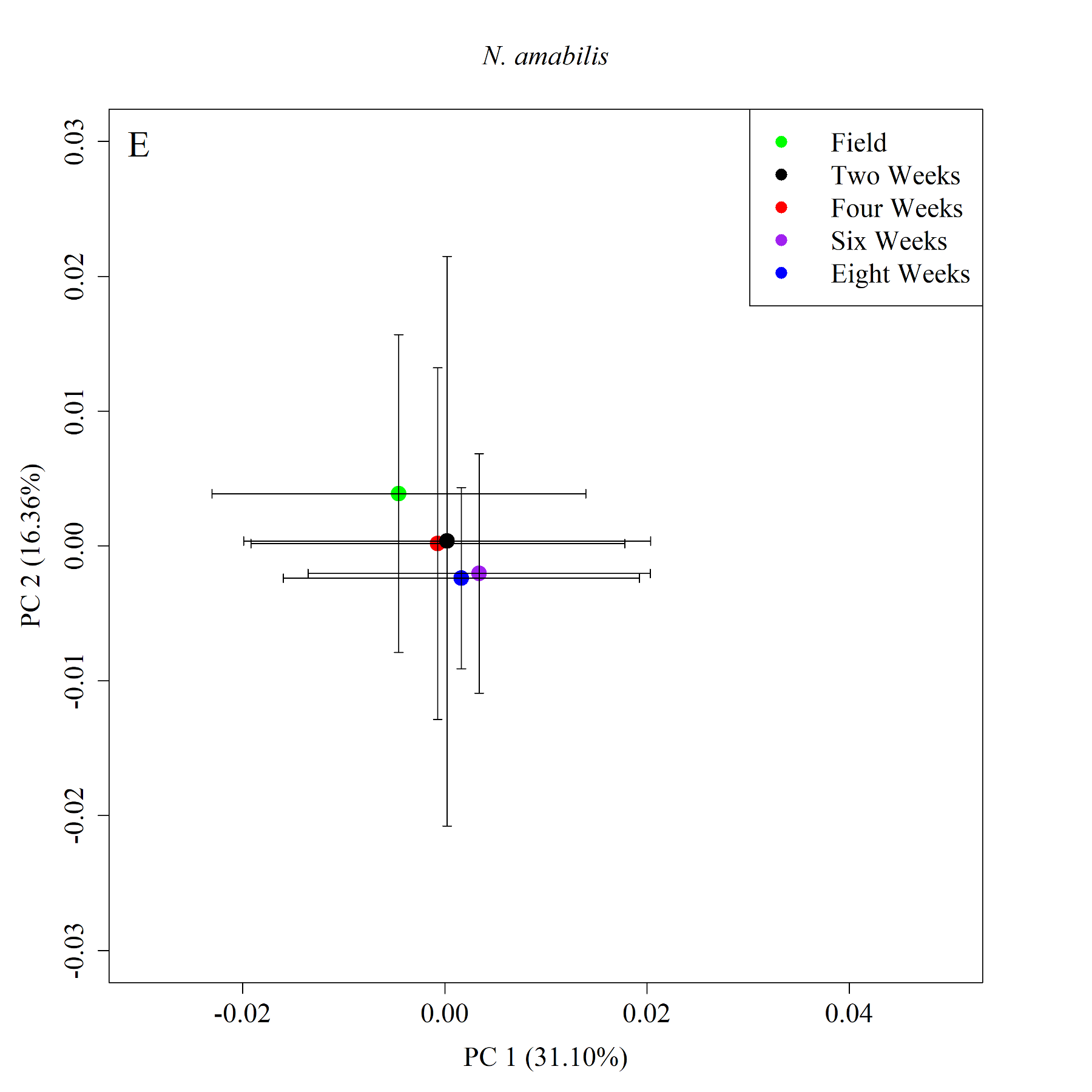

Supplement: S1 Fig — Mean of each principle components axis 1 and 2 for each species: A) C. venusta, B) G. geiseri, C) C. lutrensis, D) M. marconis, E) N. amabilis, F) N. chalybaeus, G) P. apristis, H) P. carbonaria, I) E. spectabile. Error bars represent one standard deviation. (ZIP) [file pone.0213915.s008.zip › Supplemental Fig1e.tif]

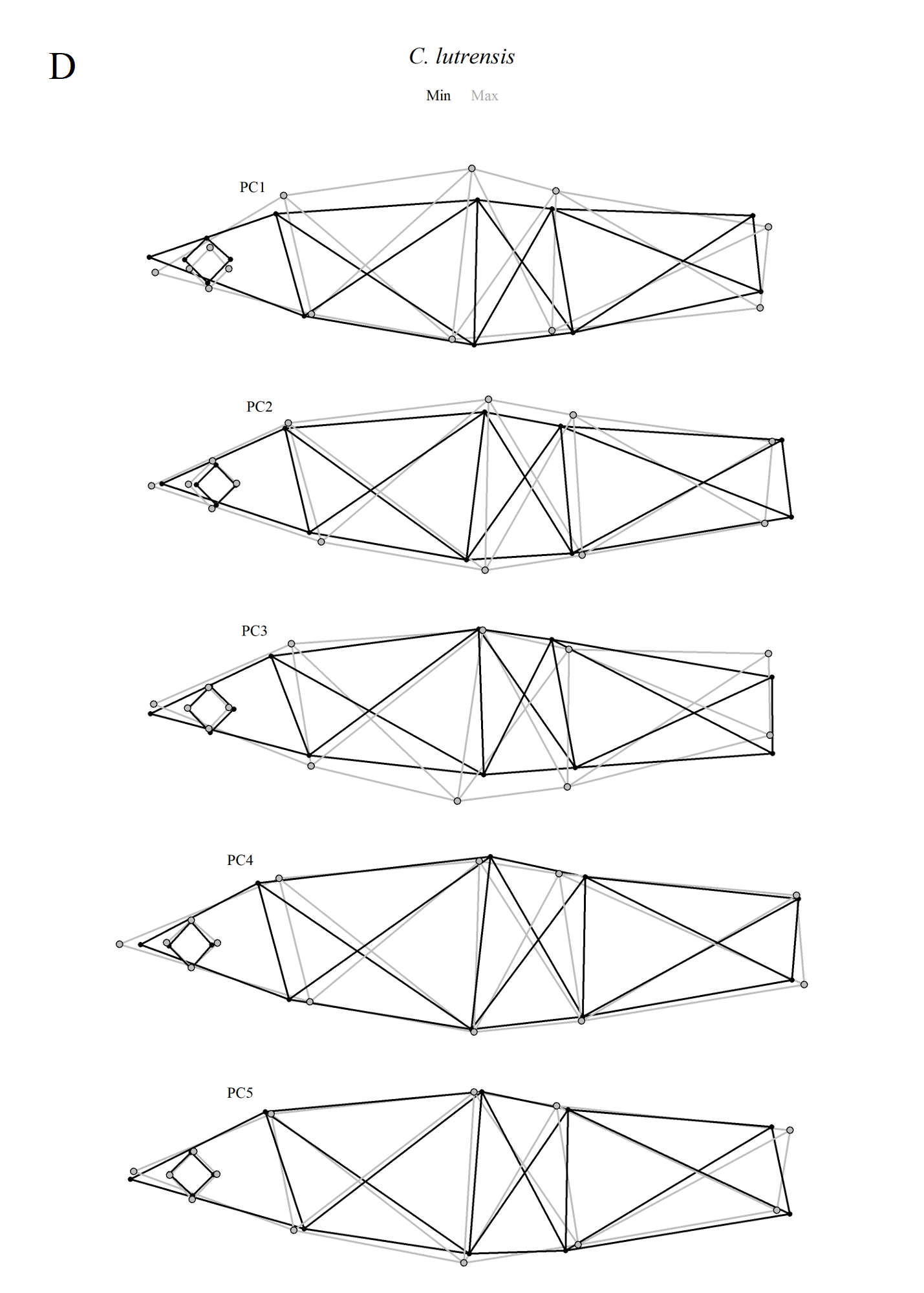

Supplement: S2 Fig — Shape plots of minimum (black lines and points) and maximum (dark gray lines and points) PC axis values for PC 1–5 for each species. A) C. venusta, B) G. geiseri, C) M. hyostoma, D) C. lutrensis, E) M. marconis, F) N. amabilis, G) N. chalybaeus, H) P. apristis, I) P. carbonaria, J) E. spectabile. (ZIP) [file pone.0213915.s009.zip › Supplemental Fig2d.tif]

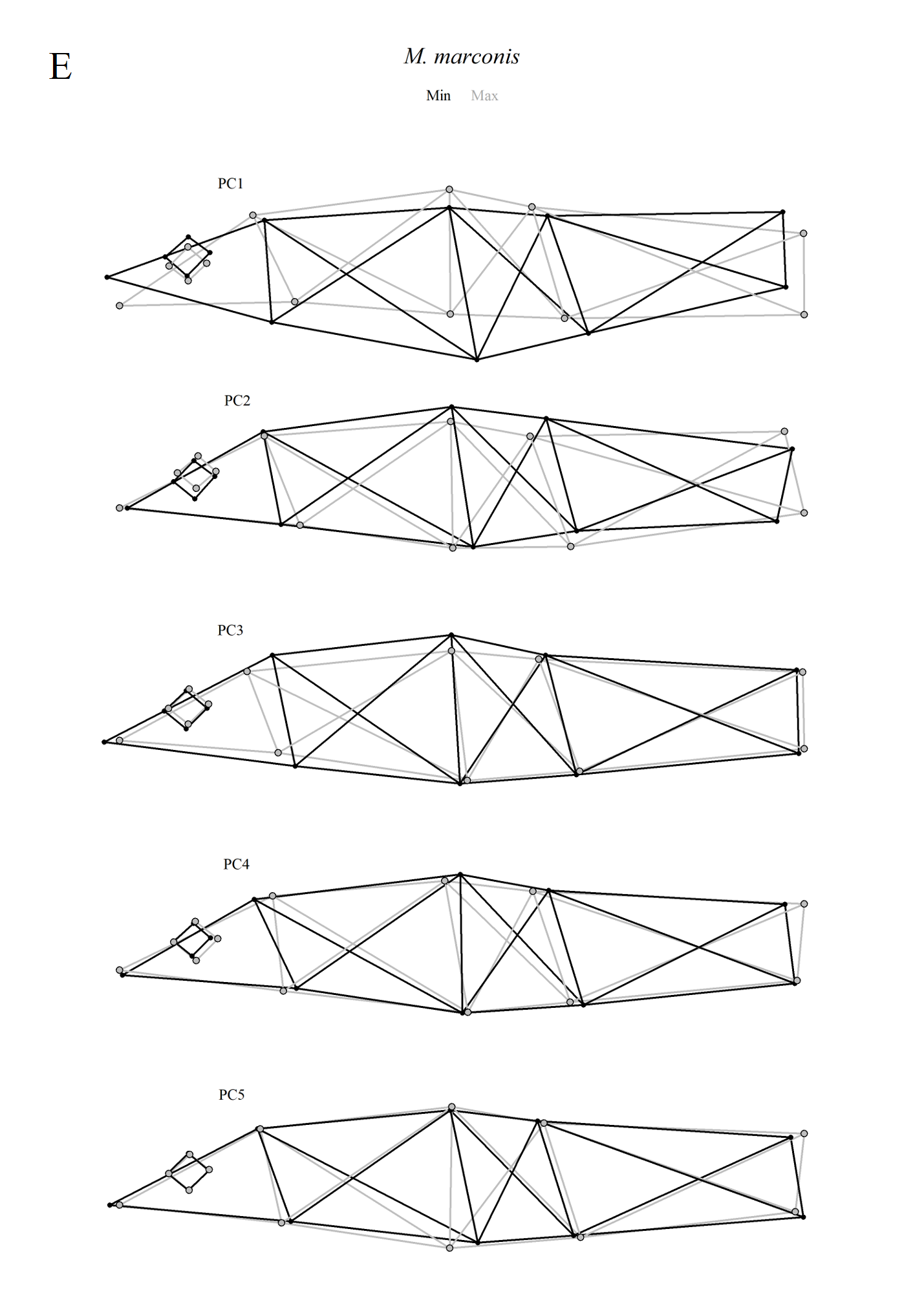

Supplement: S2 Fig — Shape plots of minimum (black lines and points) and maximum (dark gray lines and points) PC axis values for PC 1–5 for each species. A) C. venusta, B) G. geiseri, C) M. hyostoma, D) C. lutrensis, E) M. marconis, F) N. amabilis, G) N. chalybaeus, H) P. apristis, I) P. carbonaria, J) E. spectabile. (ZIP) [file pone.0213915.s009.zip › Supplemental Fig2e.tif]

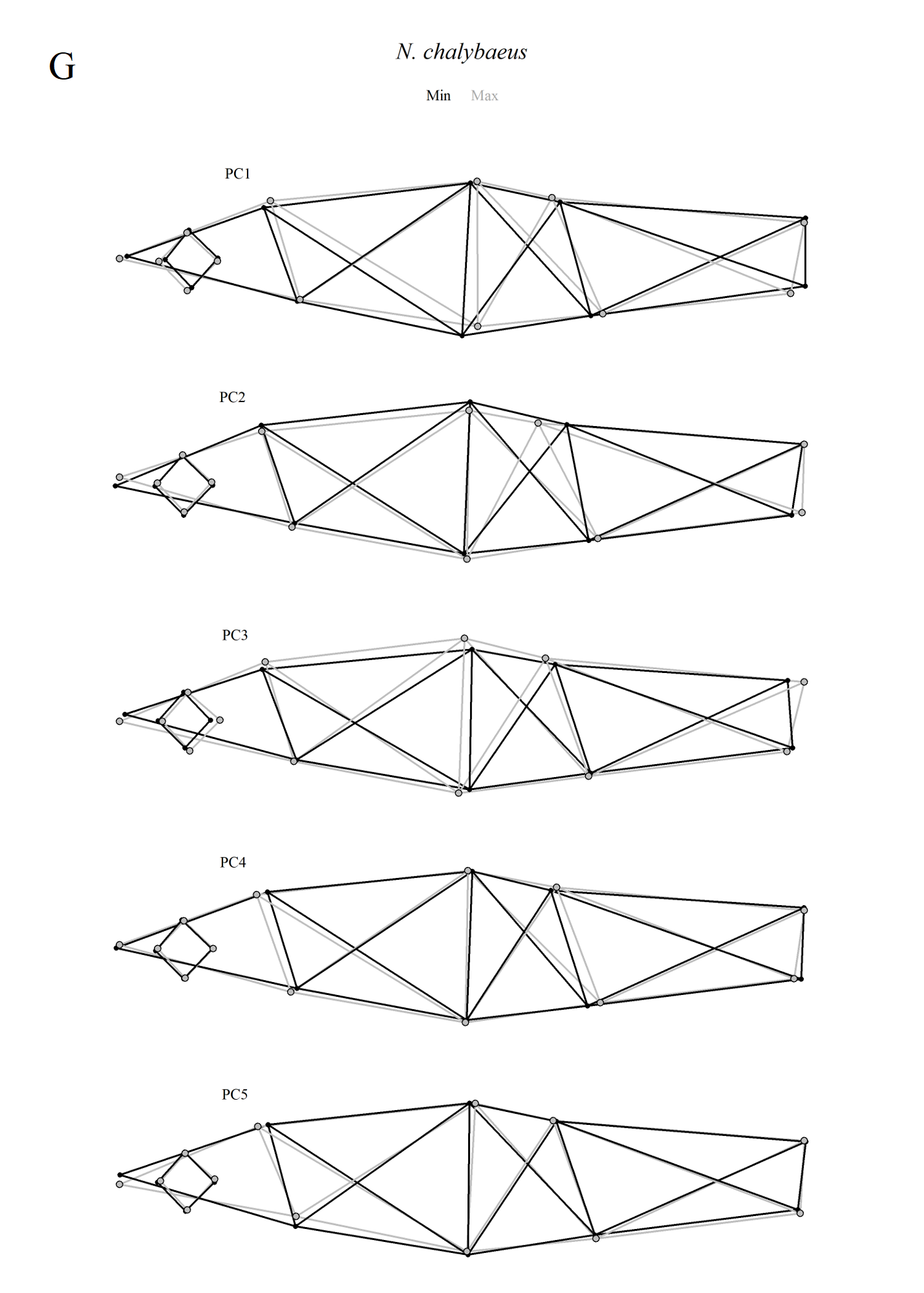

Supplement: S2 Fig — Shape plots of minimum (black lines and points) and maximum (dark gray lines and points) PC axis values for PC 1–5 for each species. A) C. venusta, B) G. geiseri, C) M. hyostoma, D) C. lutrensis, E) M. marconis, F) N. amabilis, G) N. chalybaeus, H) P. apristis, I) P. carbonaria, J) E. spectabile. (ZIP) [file pone.0213915.s009.zip › Supplemental Fig2g.tif]

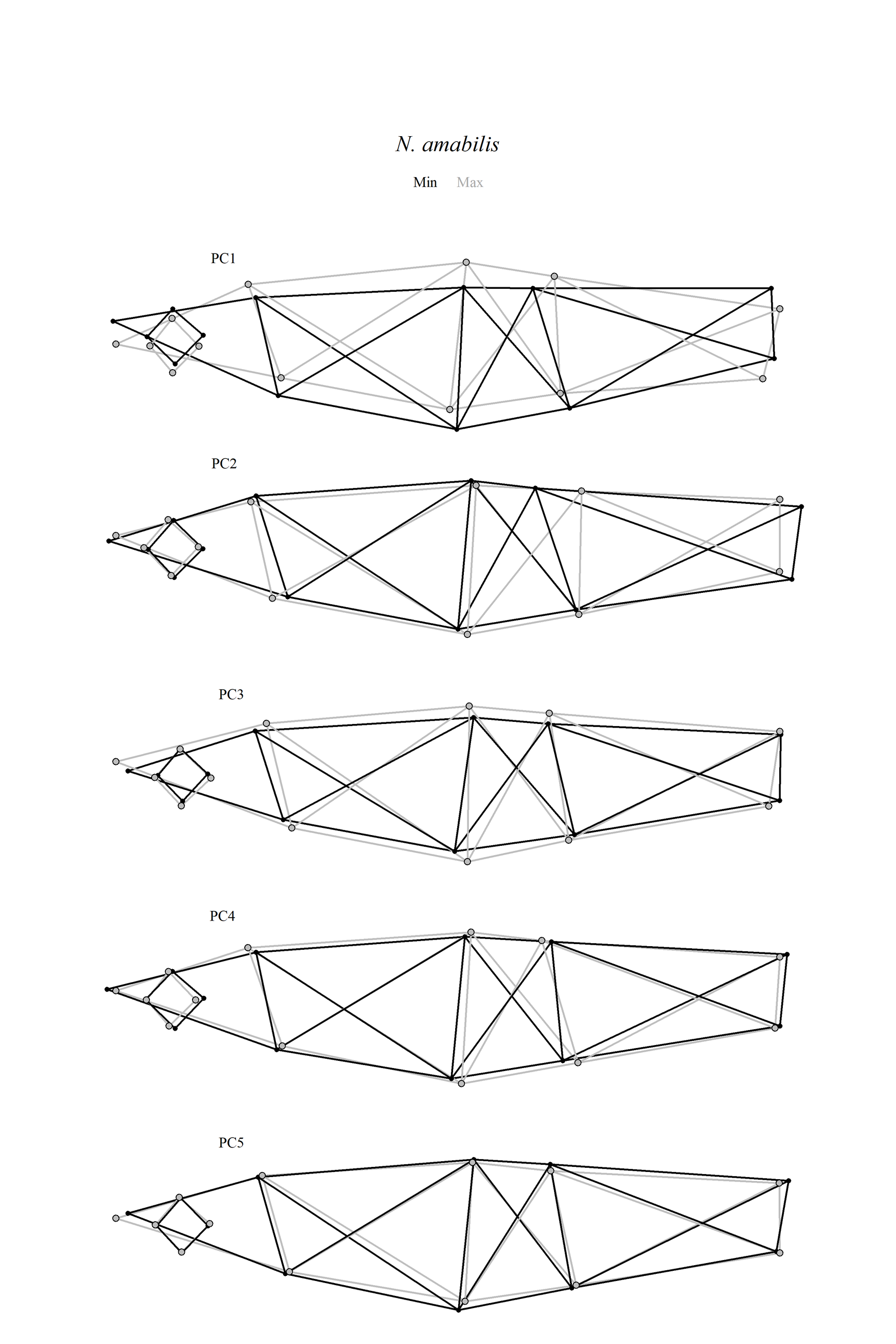

Supplement: S2 Fig — Shape plots of minimum (black lines and points) and maximum (dark gray lines and points) PC axis values for PC 1–5 for each species. A) C. venusta, B) G. geiseri, C) M. hyostoma, D) C. lutrensis, E) M. marconis, F) N. amabilis, G) N. chalybaeus, H) P. apristis, I) P. carbonaria, J) E. spectabile. (ZIP) [file pone.0213915.s009.zip › Supplemental Fig2f.tif]

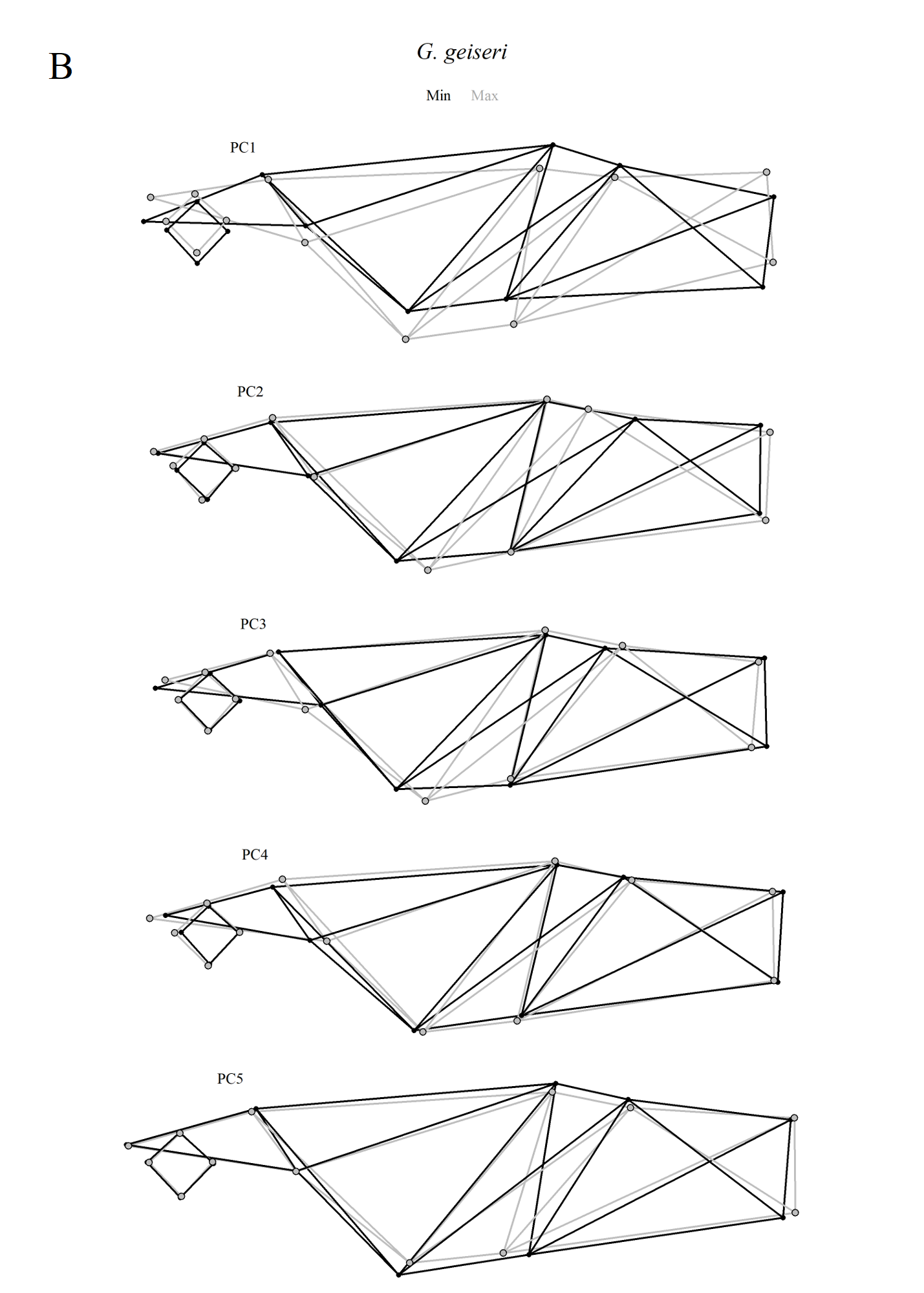

Supplement: S2 Fig — Shape plots of minimum (black lines and points) and maximum (dark gray lines and points) PC axis values for PC 1–5 for each species. A) C. venusta, B) G. geiseri, C) M. hyostoma, D) C. lutrensis, E) M. marconis, F) N. amabilis, G) N. chalybaeus, H) P. apristis, I) P. carbonaria, J) E. spectabile. (ZIP) [file pone.0213915.s009.zip › Supplemental Fig2b.tif]

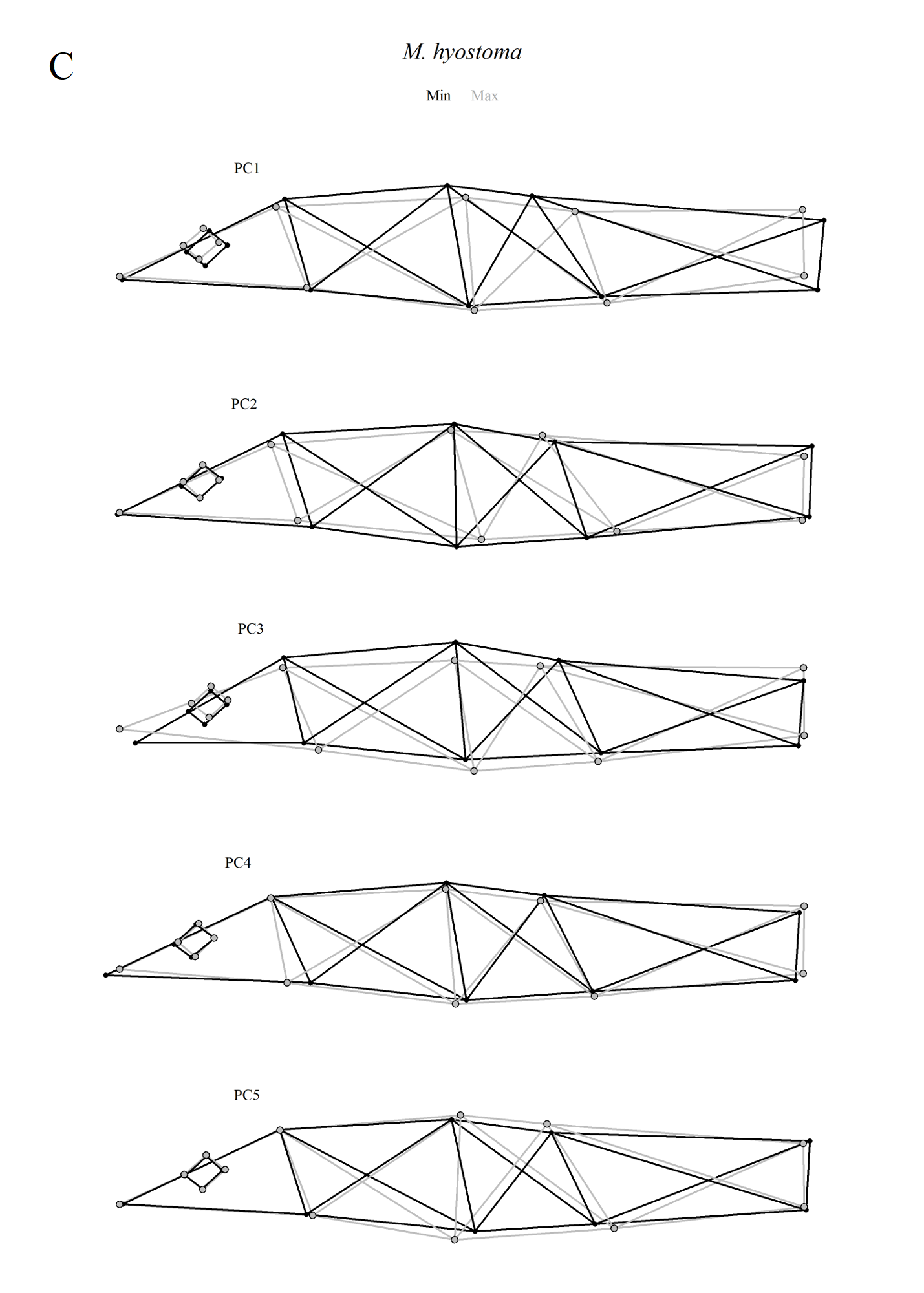

Supplement: S2 Fig — Shape plots of minimum (black lines and points) and maximum (dark gray lines and points) PC axis values for PC 1–5 for each species. A) C. venusta, B) G. geiseri, C) M. hyostoma, D) C. lutrensis, E) M. marconis, F) N. amabilis, G) N. chalybaeus, H) P. apristis, I) P. carbonaria, J) E. spectabile. (ZIP) [file pone.0213915.s009.zip › Supplemental Fig2c.tif]

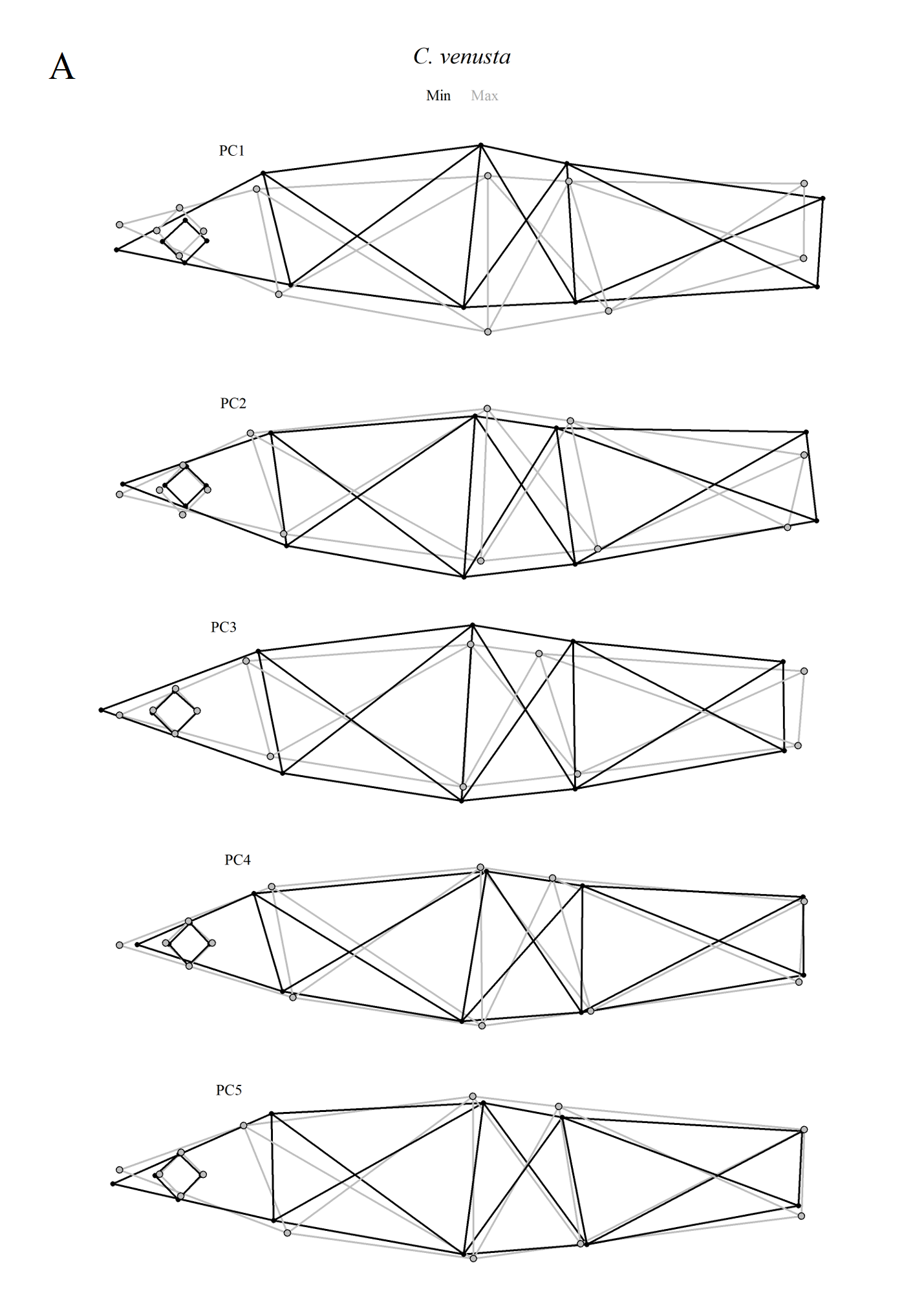

Supplement: S2 Fig — Shape plots of minimum (black lines and points) and maximum (dark gray lines and points) PC axis values for PC 1–5 for each species. A) C. venusta, B) G. geiseri, C) M. hyostoma, D) C. lutrensis, E) M. marconis, F) N. amabilis, G) N. chalybaeus, H) P. apristis, I) P. carbonaria, J) E. spectabile. (ZIP) [file pone.0213915.s009.zip › Supplemental Fig2a.tif]

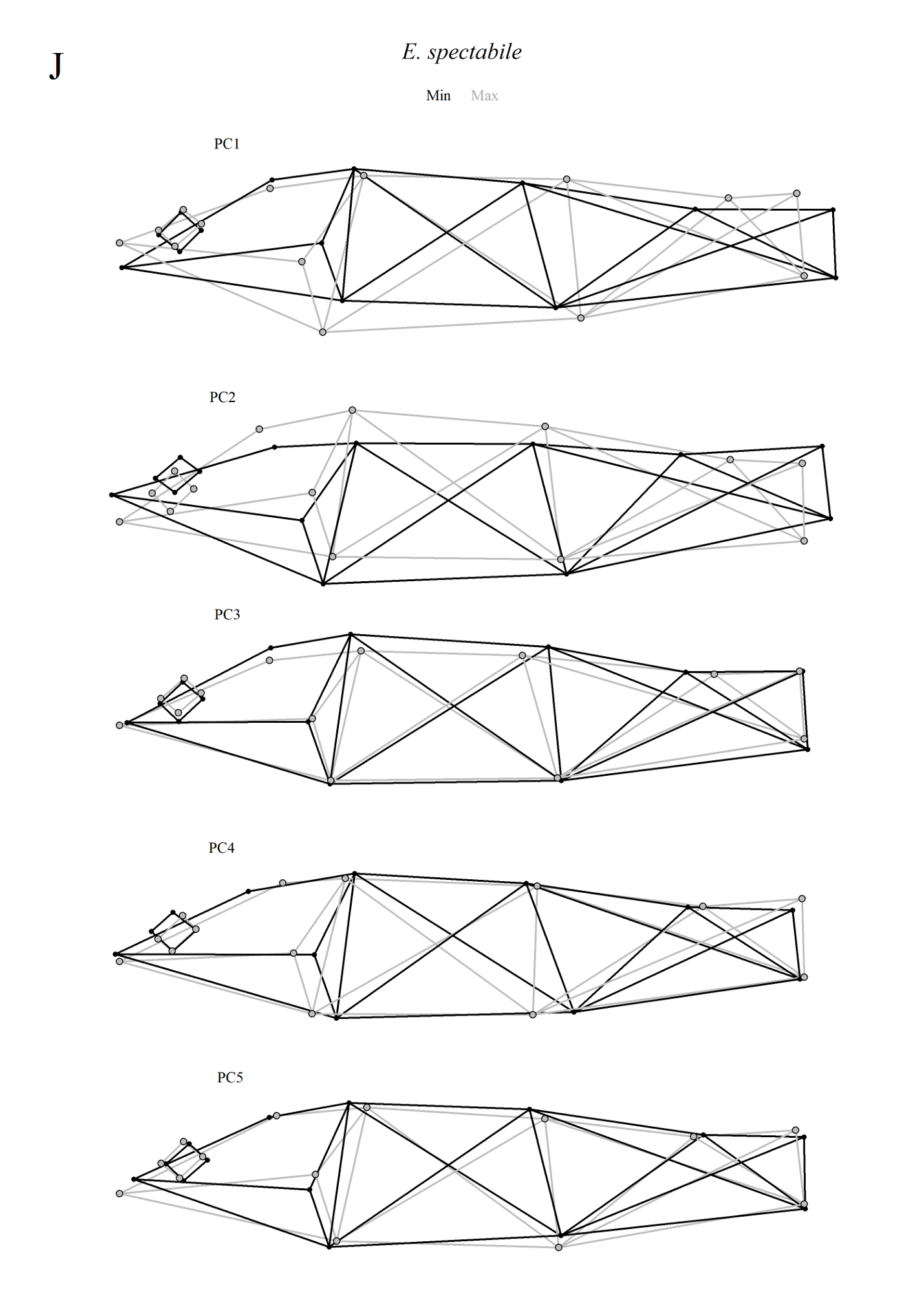

Supplement: S2 Fig — Shape plots of minimum (black lines and points) and maximum (dark gray lines and points) PC axis values for PC 1–5 for each species. A) C. venusta, B) G. geiseri, C) M. hyostoma, D) C. lutrensis, E) M. marconis, F) N. amabilis, G) N. chalybaeus, H) P. apristis, I) P. carbonaria, J) E. spectabile. (ZIP) [file pone.0213915.s009.zip › Supplemental Fig2j.tif]

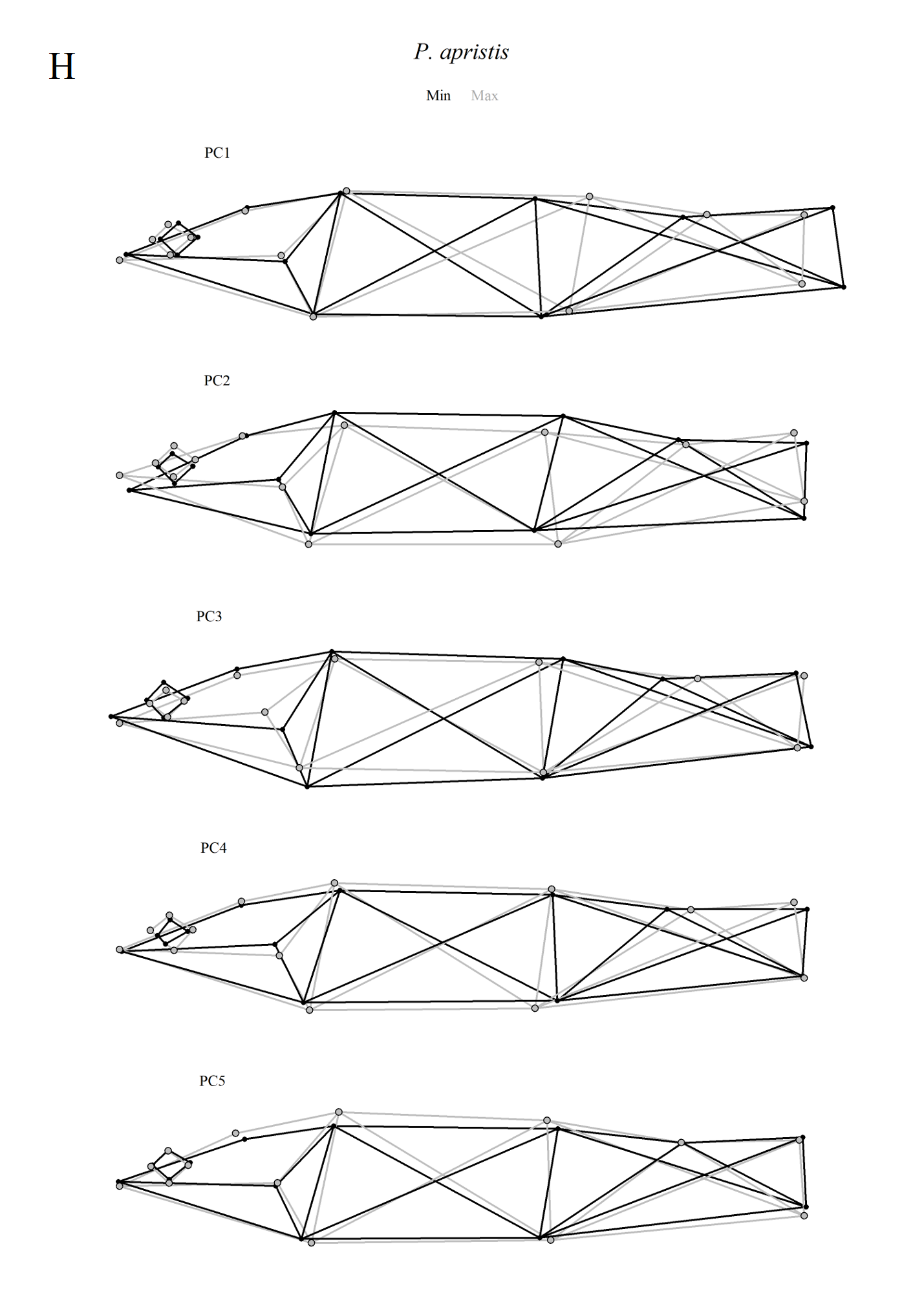

Supplement: S2 Fig — Shape plots of minimum (black lines and points) and maximum (dark gray lines and points) PC axis values for PC 1–5 for each species. A) C. venusta, B) G. geiseri, C) M. hyostoma, D) C. lutrensis, E) M. marconis, F) N. amabilis, G) N. chalybaeus, H) P. apristis, I) P. carbonaria, J) E. spectabile. (ZIP) [file pone.0213915.s009.zip › Supplemental Fig2h.tif]

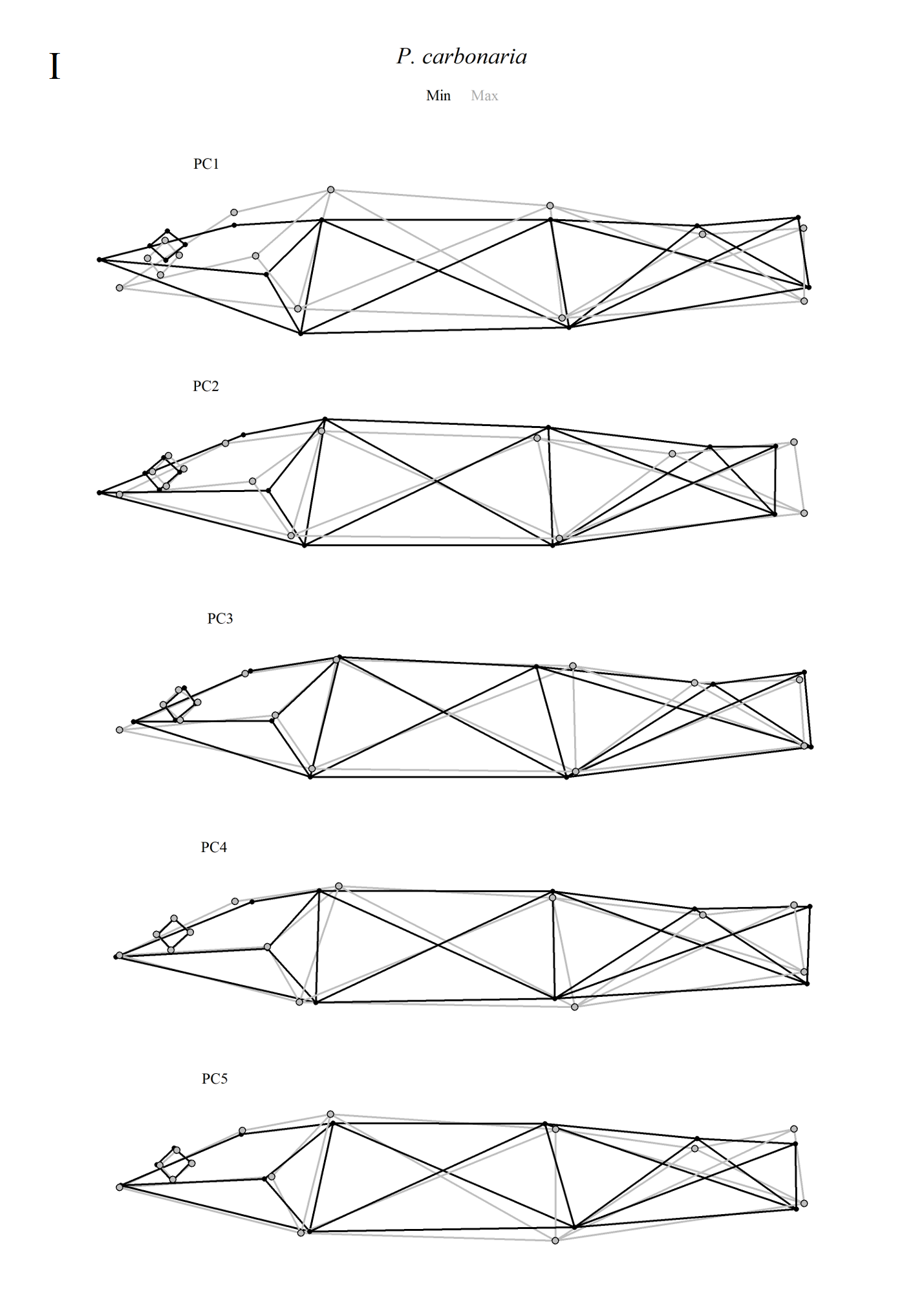

Supplement: S2 Fig — Shape plots of minimum (black lines and points) and maximum (dark gray lines and points) PC axis values for PC 1–5 for each species. A) C. venusta, B) G. geiseri, C) M. hyostoma, D) C. lutrensis, E) M. marconis, F) N. amabilis, G) N. chalybaeus, H) P. apristis, I) P. carbonaria, J) E. spectabile. (ZIP) [file pone.0213915.s009.zip › Supplemental Fig2i.tif]

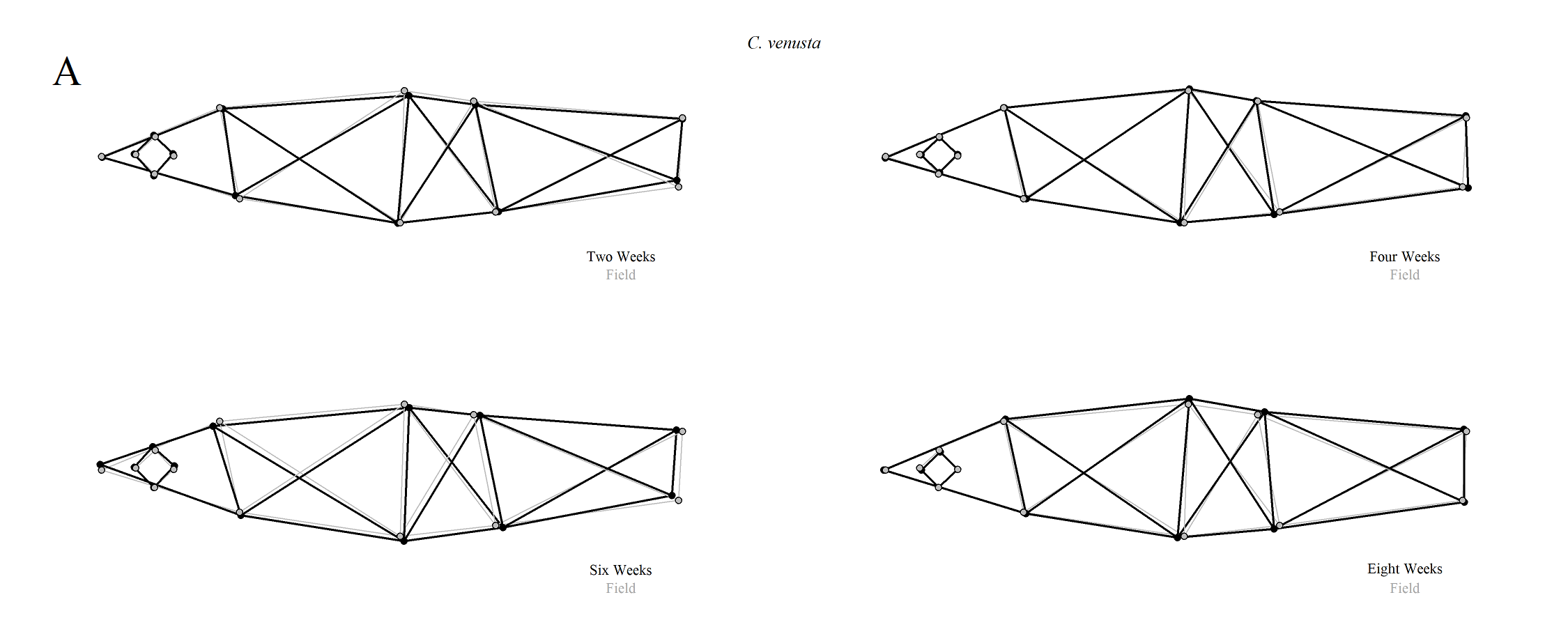

Supplement: S3 Fig — Trajectory analysis of time period relative to field for each species; shows mean shape at a particular time period (2W, 4W, 6W, and 8W) of preservation. A) C. venusta, B) G. geiseri, C) C. lutrensis, D) M. marconis, E) N. amabilis, F) N. chalybaeus, G) P. apristis, H) P. carbonaria, I) E. spectabile. (ZIP) [file pone.0213915.s010.zip › Supplemental Fig3a.tif]

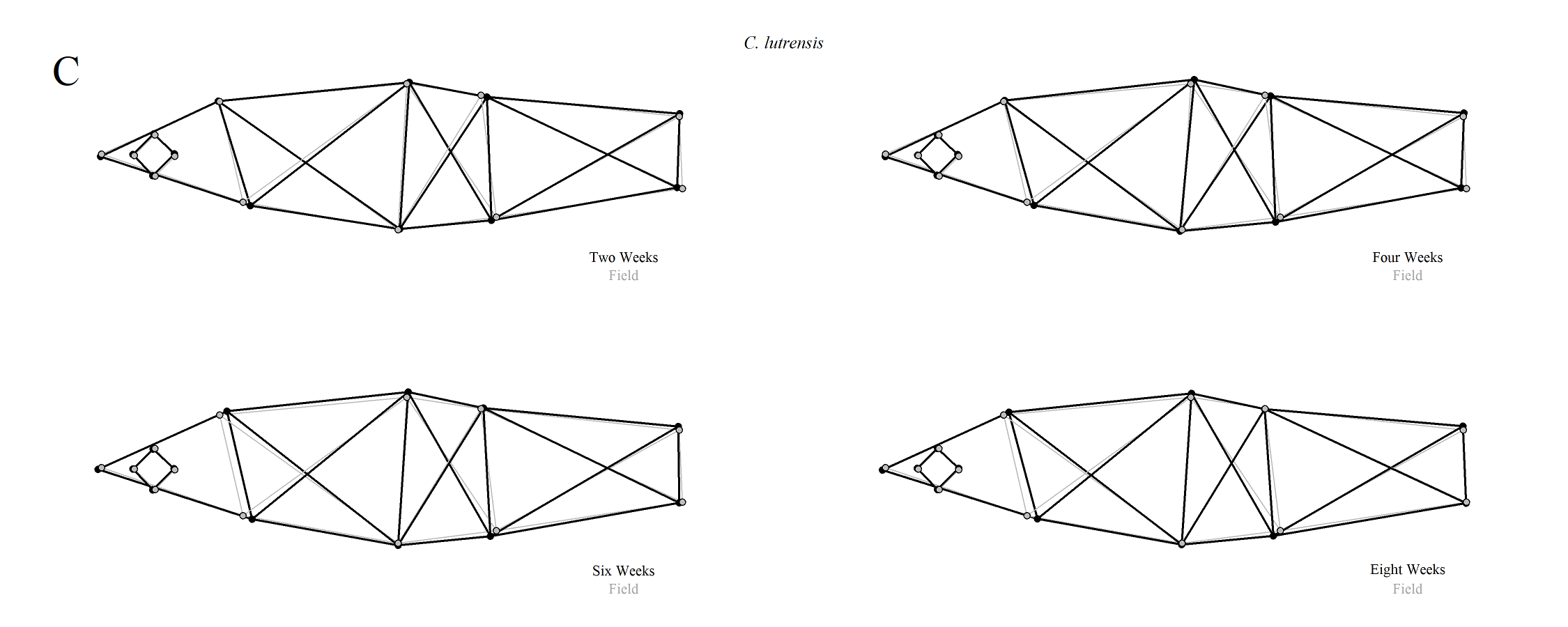

Supplement: S3 Fig — Trajectory analysis of time period relative to field for each species; shows mean shape at a particular time period (2W, 4W, 6W, and 8W) of preservation. A) C. venusta, B) G. geiseri, C) C. lutrensis, D) M. marconis, E) N. amabilis, F) N. chalybaeus, G) P. apristis, H) P. carbonaria, I) E. spectabile. (ZIP) [file pone.0213915.s010.zip › Supplemental Fig3c.tif]

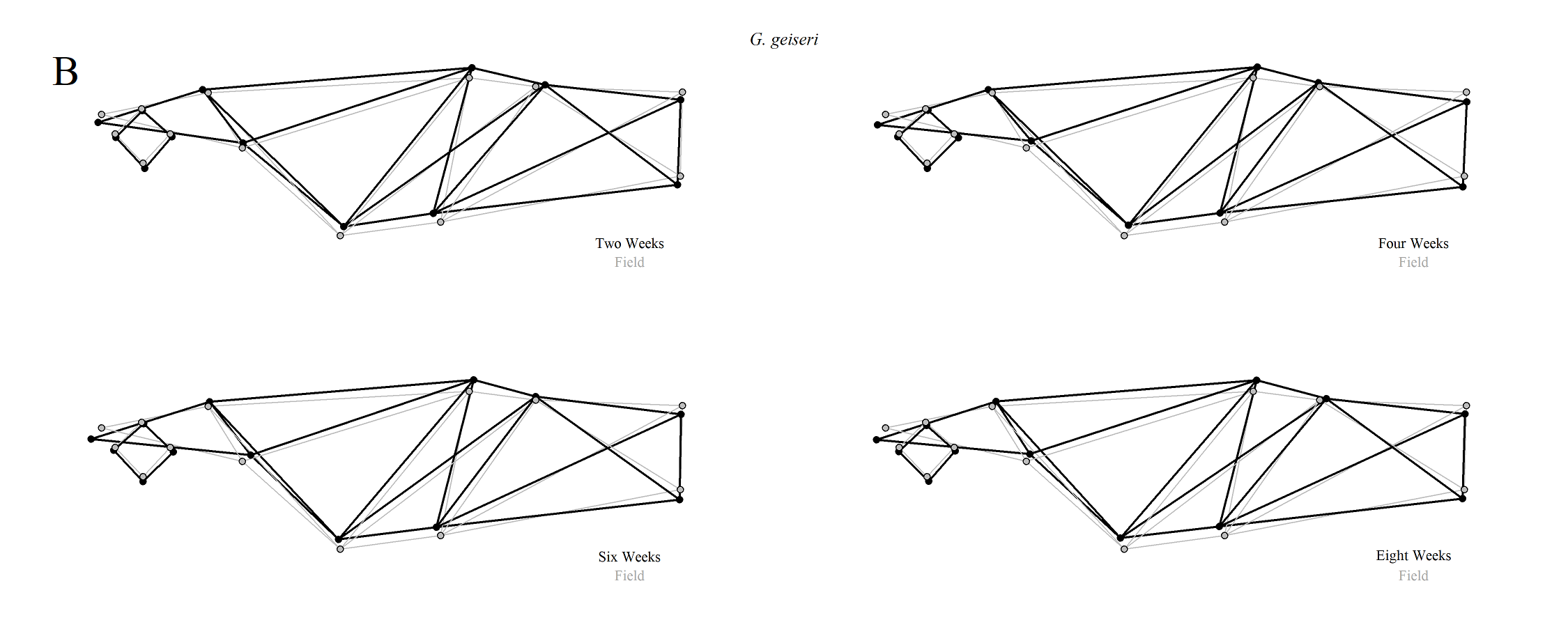

Supplement: S3 Fig — Trajectory analysis of time period relative to field for each species; shows mean shape at a particular time period (2W, 4W, 6W, and 8W) of preservation. A) C. venusta, B) G. geiseri, C) C. lutrensis, D) M. marconis, E) N. amabilis, F) N. chalybaeus, G) P. apristis, H) P. carbonaria, I) E. spectabile. (ZIP) [file pone.0213915.s010.zip › Supplemental Fig3b.tif]

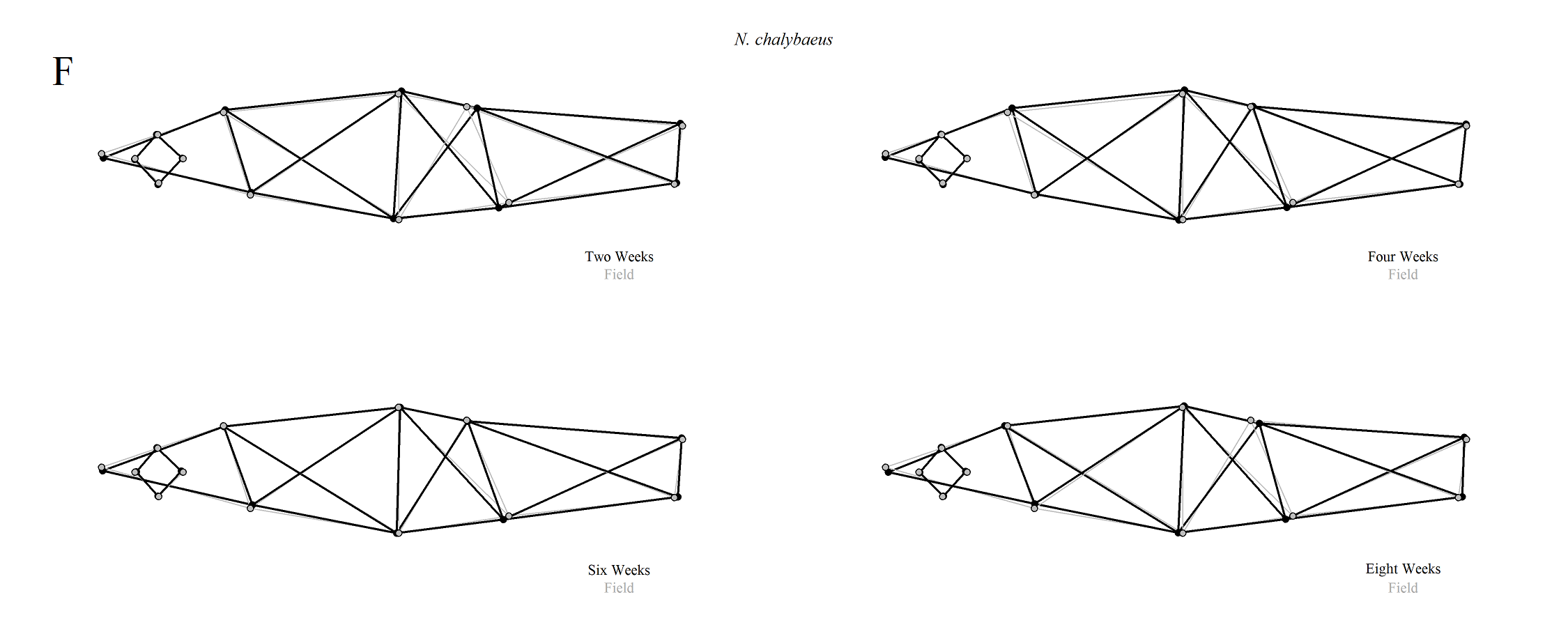

Supplement: S3 Fig — Trajectory analysis of time period relative to field for each species; shows mean shape at a particular time period (2W, 4W, 6W, and 8W) of preservation. A) C. venusta, B) G. geiseri, C) C. lutrensis, D) M. marconis, E) N. amabilis, F) N. chalybaeus, G) P. apristis, H) P. carbonaria, I) E. spectabile. (ZIP) [file pone.0213915.s010.zip › Supplemental Fig3f.tif]

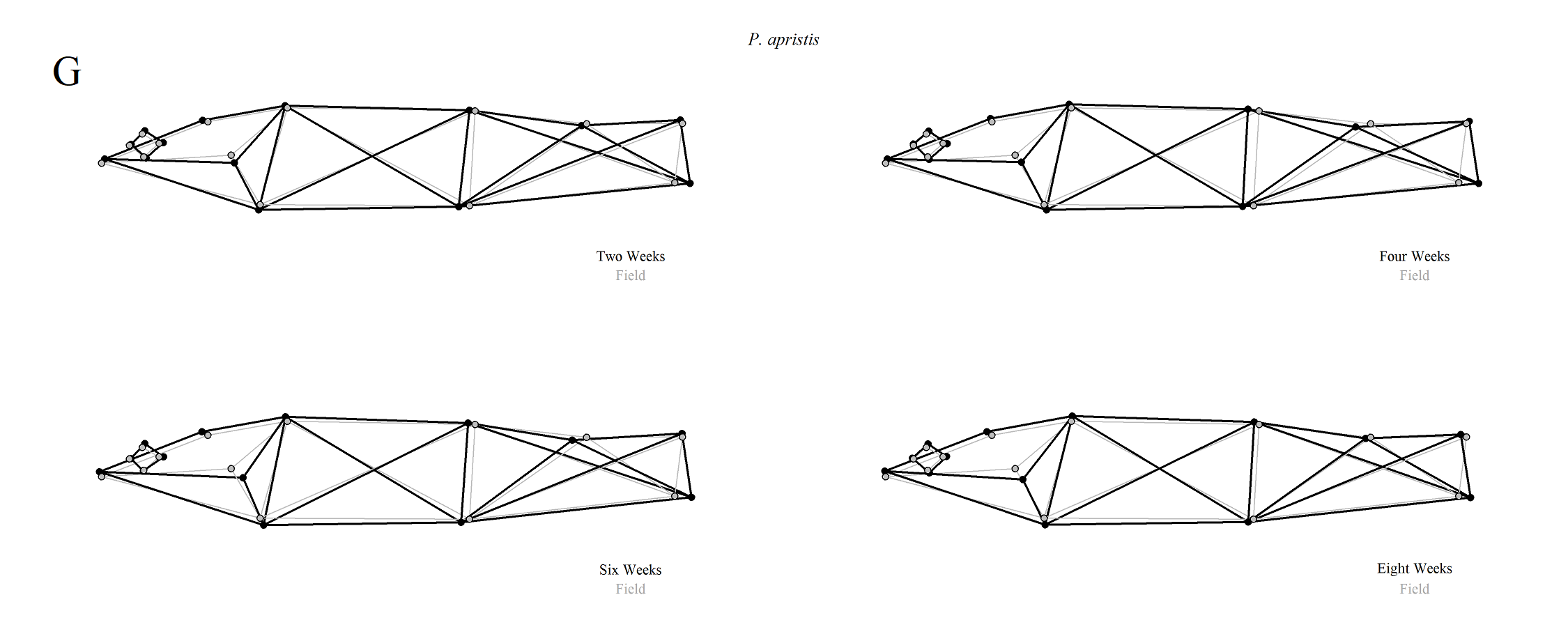

Supplement: S3 Fig — Trajectory analysis of time period relative to field for each species; shows mean shape at a particular time period (2W, 4W, 6W, and 8W) of preservation. A) C. venusta, B) G. geiseri, C) C. lutrensis, D) M. marconis, E) N. amabilis, F) N. chalybaeus, G) P. apristis, H) P. carbonaria, I) E. spectabile. (ZIP) [file pone.0213915.s010.zip › Supplemental Fig3g.tif]

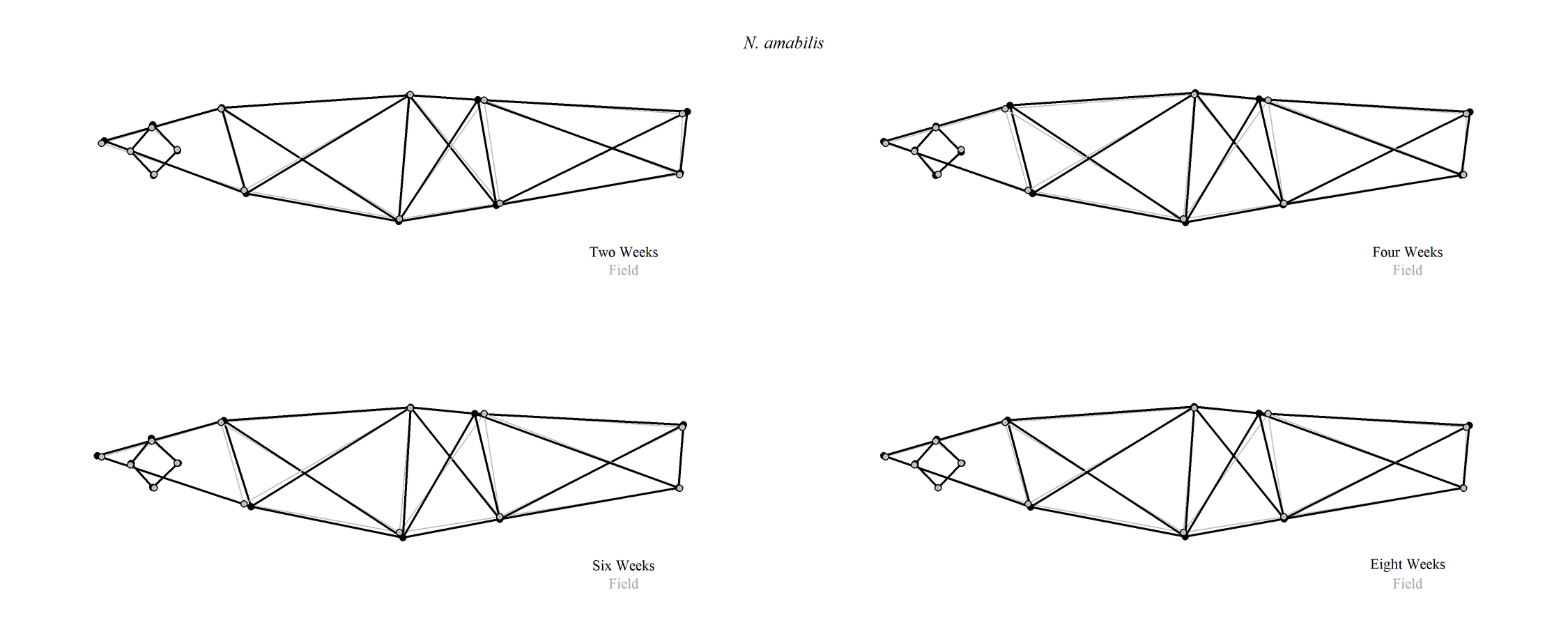

Supplement: S3 Fig — Trajectory analysis of time period relative to field for each species; shows mean shape at a particular time period (2W, 4W, 6W, and 8W) of preservation. A) C. venusta, B) G. geiseri, C) C. lutrensis, D) M. marconis, E) N. amabilis, F) N. chalybaeus, G) P. apristis, H) P. carbonaria, I) E. spectabile. (ZIP) [file pone.0213915.s010.zip › Supplemental Fig3e.tif]

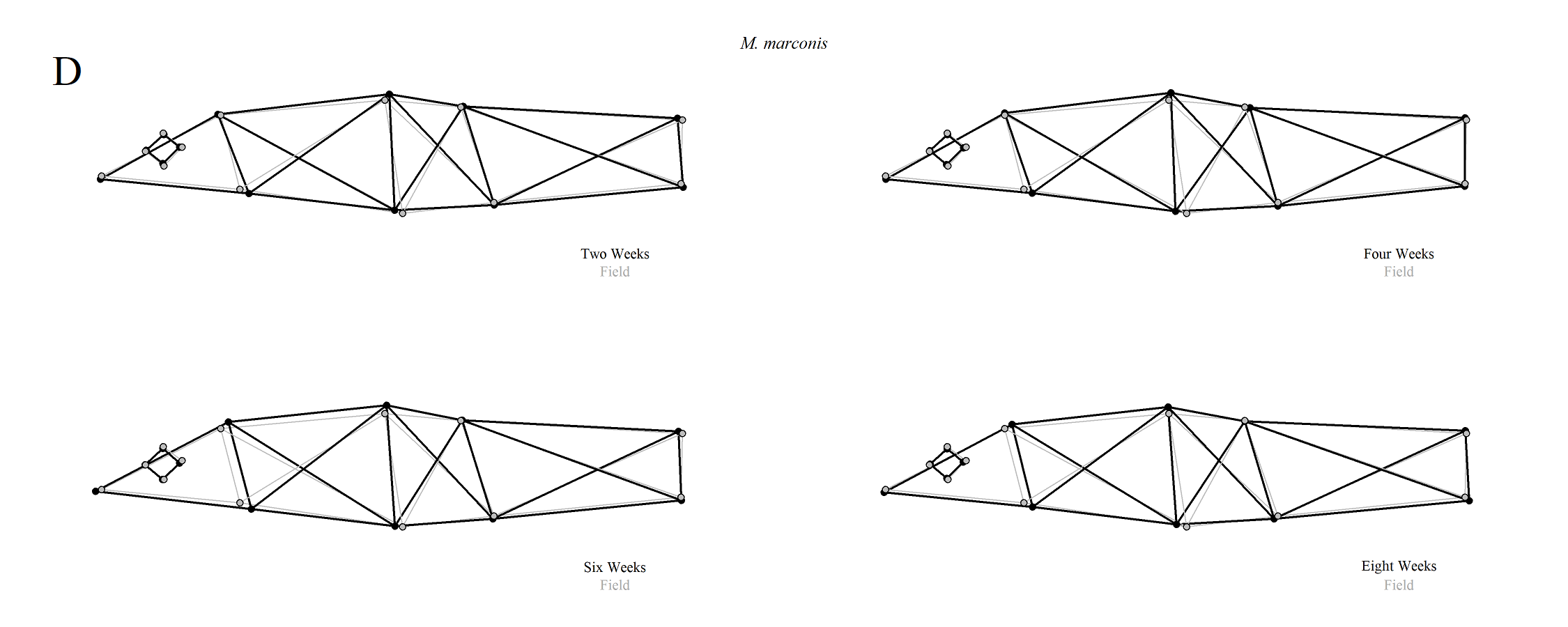

Supplement: S3 Fig — Trajectory analysis of time period relative to field for each species; shows mean shape at a particular time period (2W, 4W, 6W, and 8W) of preservation. A) C. venusta, B) G. geiseri, C) C. lutrensis, D) M. marconis, E) N. amabilis, F) N. chalybaeus, G) P. apristis, H) P. carbonaria, I) E. spectabile. (ZIP) [file pone.0213915.s010.zip › Supplemental Fig3d.tif]

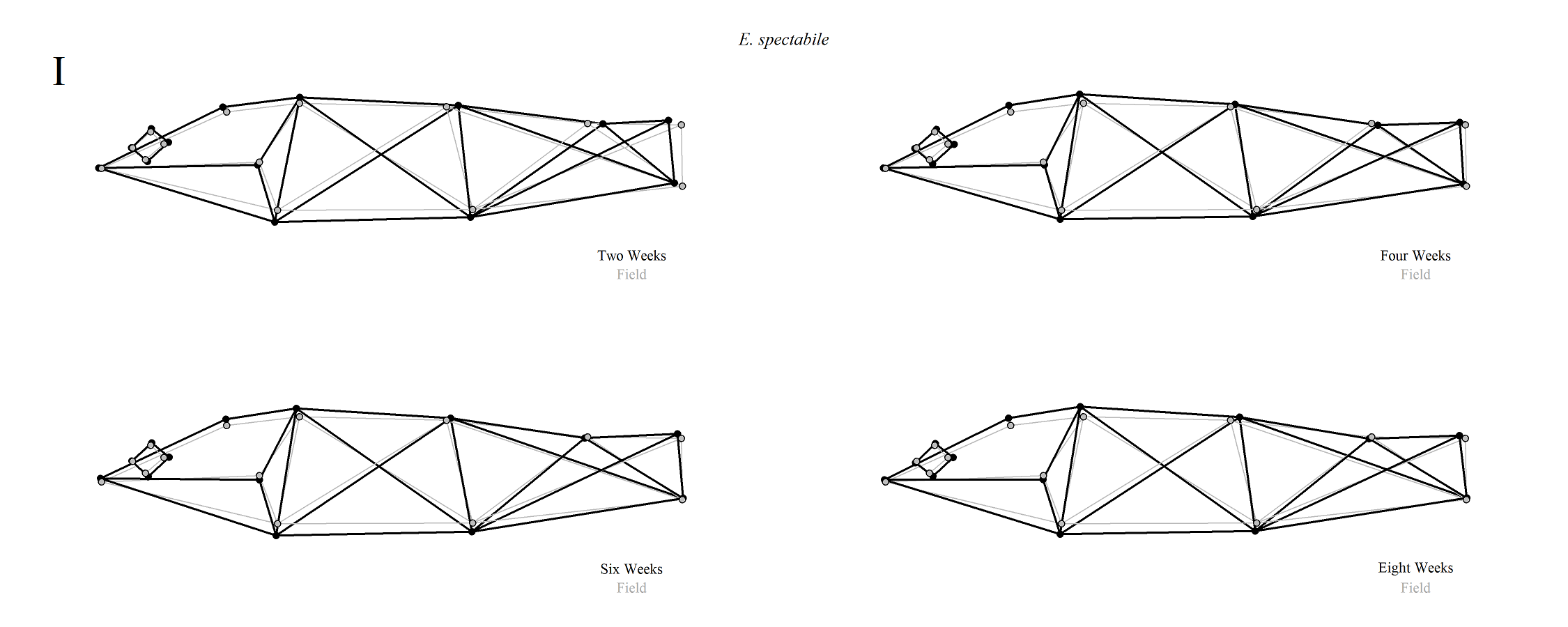

Supplement: S3 Fig — Trajectory analysis of time period relative to field for each species; shows mean shape at a particular time period (2W, 4W, 6W, and 8W) of preservation. A) C. venusta, B) G. geiseri, C) C. lutrensis, D) M. marconis, E) N. amabilis, F) N. chalybaeus, G) P. apristis, H) P. carbonaria, I) E. spectabile. (ZIP) [file pone.0213915.s010.zip › Supplemental Fig3i.tif]

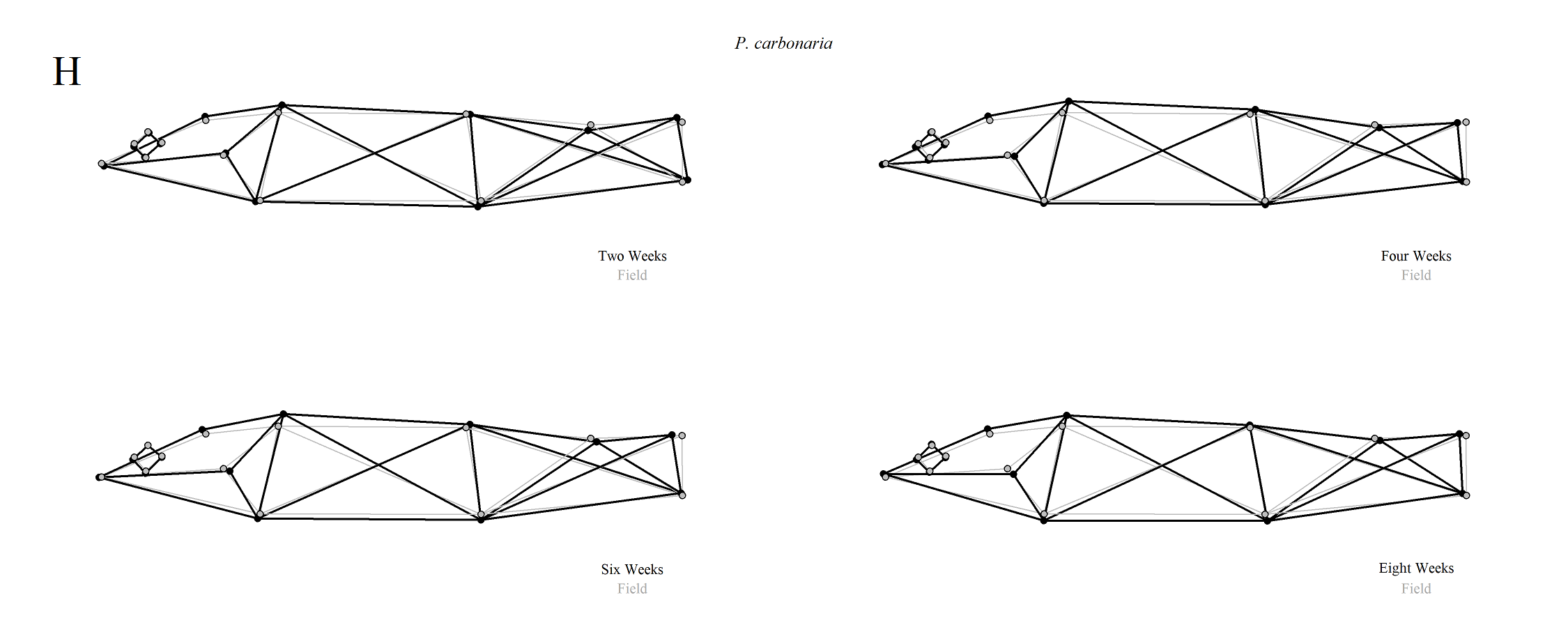

Supplement: S3 Fig — Trajectory analysis of time period relative to field for each species; shows mean shape at a particular time period (2W, 4W, 6W, and 8W) of preservation. A) C. venusta, B) G. geiseri, C) C. lutrensis, D) M. marconis, E) N. amabilis, F) N. chalybaeus, G) P. apristis, H) P. carbonaria, I) E. spectabile. (ZIP) [file pone.0213915.s010.zip › Supplemental Fig3h.tif]
